# Knowledge flows from science to AI technology: Identifying core and brokerage technological roles

**DOI:** 10.1371/journal.pone.0341005

**Published:** 2026-02-19

**Authors:** Seokhui Lee, Jisoo Hur, Junseok Hwang, Dieter F. Kogler, Keungoui Kim

**Affiliations:** 1 Technology Management, Economics and Policy Program, College of Engineering/ Integrated Major in Smart City Global Convergence, Seoul National University, Seoul, Republic of Korea; 2 Dr. Theo Schöller Chair of Technology and Innovation Management, TUM School of Management, Munchen, Germany; 3 Spatial Dynamics Lab, Regional and Urban Planning Richview Campus, University College Dublin, Belfield, Dublin, Ireland; 4 Humanities, Arts, and Social Sciences Division (HASSD), Underwood International College, Yonsei University, Seoul, Republic of Korea; Institute of Economics (Scuola Superiore Sant'Anna / RFF-CMCC European Institute on Economics and the Environment, ITALY

## Abstract

The rapid advancement of artificial intelligence (AI) technologies has not only driven convergence with diverse technological domains but also swiftly spread across various industrial sectors. As a knowledge-intensive field, AI is particularly shaped by the flow of knowledge from scientific research to technological development, yet remains insufficiently examined in a systematic and structural way. This study addresses this gap by investigating science-to-technology knowledge flow that underpins AI’s technological evolution. We propose a semantic science-technology exploration framework specifically designed for the AI domain, consisting of the two stages: technology classification and semantic topic exploration. First, AI patents are classified into four categories using centrality measures derived from a CPC co-occurrence network. Then, we extract abstracts from both patents and their cited scientific publications to apply BERTopic modelling and generate topic labels using generative AI. Analyzing AI-related patents filed from 2002 to 2021, we trace key technological trends and elucidate the structural pathways of knowledge flow science to technology. The findings offer practical implications for corporate R&D strategies and innovation policy design in the era of AI.

## 1. Introduction

The rapid advancement of Artificial Intelligence (AI) has profoundly shaped technological innovation, fueled economic growth, and driven broad societal transformation. In response, global competition to secure national leadership in AI technologies has intensified [1], and corporate efforts to establish technological dominance have expanded considerably [2]. In this context, identifying the developmental trajectories of AI and understanding the dynamics of AI-related knowledge flows have become critical for stakeholders, particularly firms and governments, seeking to capture emerging opportunities and develop informed strategic agendas.

Yet AI constitutes a highly complex and evolving technological paradigm [3], unfolding along multiple, intersecting trajectories. This complexity necessitates the integration of diverse data sources and perspectives, rather than reliance on any single dataset [4]. Accordingly, measuring and interpreting the multifaceted knowledge components underpinning AI’s development poses a significant analytical challenge.

To address this, the present study focuses on a crucial yet underexplored dimension of AI innovation: the flow of knowledge from science to technology. Even prior to the emergence of AI, the science-to-technology knowledge flow was widely recognized as a fundamental mechanism for knowledge recombination and innovation. Numerous studies conceptualize scientific publications as proxies for scientific knowledge and patents as proxies for technological output, interpreting citations of scientific articles in patents as evidence of scientific knowledge being transformed into technological applications [5,6]. Notably, patents grounded in scientific knowledge have been found to exert a stronger influence on subsequent technological advancements [7–9], highlighting the science-to-technology knowledge flow as a key driver of innovation. However, this pattern is far from uniform. The intensity and nature of science-to-technology linkages vary considerably across industries [10–12], technological domains [13], and organizational contexts [14]. These variations suggest that different industries draw upon and apply scientific knowledge based on their own evaluative criteria and internal logic [7], selectively leveraging science to generate technological innovation.

In the case of AI, this science-to-technology flow is particularly critical. AI is fundamentally rooted in scientific experimentation and hypothesis testing [15] and represents a knowledge-intensive domain that integrates insights from multiple academic disciplines to enable technological realization [16–18]. Nevertheless, most existing studies have examined the scientific and technological dimensions of AI in isolation, neglecting their co-evolution. Despite this disconnect, recent research has revealed parallel trends in both domains: an initial focus on foundational research has increasingly shifted toward applied technologies and the broader diffusion of AI applications [18,19]. In particular, machine learning, deep learning, and network-based approaches have emerged as dominant themes across both science and technology landscapes [20,21]. While these studies hint at a co-evolutionary pattern, few have systematically examined the underlying science-to-technology knowledge flows that shape AI’s evolution. This study aims to fill that gap by analyzing how scientific knowledge contributes to the development of AI technologies over time.

To this end, we propose a science–technology exploration framework specifically designed for the AI domain. First, we categorize AI-related patents into four groups using centrality metrics derived from a Cooperative Patent Classification (CPC) co-occurrence network, enabling us to distinguish core technologies that lead the ecosystem and those that function as brokers facilitating convergence [22]. We then extract abstracts from both patents and their cited scientific articles, apply BERTopic modeling to identify thematic structures, and generate interpretable topic labels using generative AI techniques [23]. Focusing on AI patents filed between 2002 and 2021, we trace the structural trajectories of science-to-technology knowledge flows across the identified technology categories.

Our findings reveal distinct patterns of knowledge flow depending on the structural role of the technology. Technologies exhibiting a brokerage function, those that connect disparate fields, tend to utilize scientific knowledge selectively and contextually, in response to specific technological demands. In contrast, technologies with low brokerage characteristics follow more science-driven development paths, with scientific input playing a more direct and foundational role. Furthermore, methodologies and performance-enhancing innovations within AI itself have emerged as central technological domains, acting as primary conduits for the transfer of scientific knowledge into technological applications.

This study underscores that even within a single technological field such as AI, knowledge flow patterns vary significantly according to the structural positioning of technologies. By applying our framework to this rapidly evolving domain, we contribute to the literature on knowledge flows and innovation systems, while offering a detailed perspective on the interplay between science and technology in AI. Practically, our findings provide valuable insights for firms, policymakers, and other stakeholders, helping them assess their position within the AI innovation ecosystem and devise strategies that bridge scientific discovery and technological implementation.

The remainder of this paper is organized as follows. Section 2 reviews prior research on science-to-technology knowledge flows and recent trends in AI technological development. Section 3 outlines the research methodology. Section 4 presents the findings of our knowledge flow analysis across different AI technology categories, and Section 5 discusses the theoretical and practical implications of the study.

## 2. Literature review

### 2.1. Knowledge from science to technology

The relationship between science and technology has long been a central topic in innovation studies. Science is commonly defined as the systematic accumulation of knowledge through coherent theoretical frameworks and experimental validation, while technology is characterized by the practical application of this knowledge through engineering and industrial practices [24]. In many contexts, technology relies heavily on tacit or rule-based knowledge. However, scientific knowledge is often introduced to overcome technical limitations [25], aligning with the linear model of innovation, in which scientific discoveries lead to engineering solutions and eventually to technological advances [26]. The stepwise transformation of novel scientific ideas into practical applications is widely considered a core mechanism of innovation [27].

Recent studies increasingly emphasize the importance of scientific knowledge as a key resource for the recombination of knowledge in technological development, highlighting the flow of knowledge from science to technology. Patents that cite scientific publications are widely regarded as tangible evidence of the translation of scientific discoveries into technological inventions [5,6], signifying the materialization of abstract scientific insights into practical outcomes [11]. Notably, patents that cite scientific literature tend to receive more forward citations and exert greater influence within their respective technological domains [7]. As such, the number of scientific references a patent makes has become a widely accepted proxy for the technological impact of scientific research [8].

Technologies grounded in scientific knowledge are particularly associated with exploratory innovation, serving as an effective means of generating novel knowledge components [9]. These patents also tend to have higher transaction success rates and command greater value in the market for technology [28]. For instance, an empirical study by Chen et al. [29] on Taiwanese electronics firms found that patents with a higher number of scientific citations were significantly associated with increased Total Factor Productivity, highlighting the substantial contribution of academic research to firm-level innovation. Moreover, science-based patents have been shown to facilitate green technology innovation and contribute positively to sustainability outcomes [30].

However, the presence of scientific citations alone does not necessarily imply high technological value. Meyer [31] argued that modern patent-to-paper citations often reflect complex and multi-dimensional interactions between science and technology, rather than simple, linear relationships. This perspective underscores the need for more in-depth, qualitative analyses that consider the purpose and context of citations, rather than relying solely on citation counts. As the number of patents citing scientific literature continues to grow [32,33], there is a growing call for semantic-level analyses of science-to-technology knowledge flows.

The impact of these knowledge flows depends on both the characteristics of the cited scientific work and the absorptive capacity of the recipient organization. Poege et al. [7], for example, found that high-quality scientific publications are more likely to be cited in patents and are associated with higher market value. This suggests that firms selectively leverage scientific knowledge that aligns with their internal evaluation criteria and strategic priorities. The ability of inventors to strategically recombine scientific knowledge has been identified as a critical factor in fostering technological novelty [34]. Consequently, the patterns and impacts of science–technology linkages can vary considerably across national and organizational contexts [14].

In addition to organizational factors, the nature of science-to-technology knowledge flows also varies across technological and industrial sectors. Roach and Cohen [10] found that industries such as biotechnology, pharmaceuticals, and aerospace exhibit a high dependency on scientific research for their R&D and patenting activities. In contrast, more traditional manufacturing industries, such as automobiles, metals, and electrical equipment, tend to rely less on scientific inputs. Similarly, Kong et al. [12] reported that sectors such as biotechnology, biological materials, pharmaceuticals, and food chemistry demonstrate strong linkages to science, whereas other industries more frequently build on existing technological knowledge. From a scientific disciplinary perspective, Ahmadpoor and Jones [11] found that life sciences and nanotechnology exhibit strong and direct science–technology linkages, while fields like mathematics and astronomy show weaker connections, with computer science occupying an intermediate position.

In summary, scientific knowledge forms a foundational basis for technological innovation, but the pathways through which it flows into technology vary depending on both technological domains and organizational contexts. Understanding how scientific knowledge is transferred, adapted, and transformed into technological outcomes is essential for advancing our understanding of the innovation process. This study focuses on analyzing these science-to-technology knowledge flows in the rapidly evolving field of AI. Specifically, we classify AI technologies and investigate how knowledge flow patterns differ across the resulting categories.

### 2.2. AI science and technology

The development of artificial intelligence (AI) technologies is fundamentally grounded in scientific knowledge, and understanding the flow of knowledge from science to technology in this domain provides critical insights into AI’s evolutionary dynamics and a foundation for identifying future innovation opportunities [18,35]. Accordingly, this section reviews key studies that examine the structural linkages between science and technology in the AI field.

It is important to consider whether distinguishing between the scientific and technological dimensions of AI is meaningful, and whether tracing the knowledge flow between them yields valuable insights. This question lies at the heart of understanding the nature of AI itself. AI systems are not merely technical artifacts; rather, they are grounded in scientific hypotheses that propose intelligent behavior can emerge from specific computational architectures and mechanisms [15]. As a result, modern AI is widely recognized as a knowledge-intensive field that translates scientific theories into real-world applications [18]. Foundational disciplines such as mathematics, physics, and biology have made substantial contributions to AI by providing both theoretical underpinnings and algorithmic innovations [17]. Notably, neuroscience has had a particularly influential impact on AI research and continues to serve as a key source of inspiration for future advancements [16]. For instance, core AI techniques such as deep learning and reinforcement learning are based on mathematical principles including Contrastive Divergence, the Markov Process, and the Bellman Equation [17].

Recognizing the scientific foundations of AI, an increasing number of studies have sought to analyze the research trends that constitute its knowledge base. These studies commonly follow the prevailing convention of representing scientific activity through academic publications and technological activity through patents [35]. Consequently, much of the existing literature on AI trend analysis has focused primarily on scientific publications. For example, Qian et al. [36] analyzed a large corpus of AI research papers to construct a hierarchical map of the field’s evolution, identifying artificial neural networks as the dominant paradigm. Given the rapid pace of AI development, conference proceedings have played a particularly important role in the dissemination of knowledge. Reflecting this trend, Wibawa et al. [21] analyzed the titles of conference papers to identify emerging technological themes. While early AI research was largely theoretical in nature, the field has increasingly shifted toward applied and solution-oriented development [37]. Moreover, AI research has become progressively interdisciplinary, as demonstrated by longitudinal studies tracking its expansion into a wider array of scientific domains over recent decades [38,39].

Parallel patterns have been observed in patent-based studies. Early AI patents predominantly focused on foundational techniques and algorithmic innovations, whereas more recent patents emphasize practical applications and industrial convergence [19]. Some studies have employed citation network analysis to trace technological trends [40], while others have applied natural language processing techniques to patent abstracts to identify emerging topics [41] or examined corporate patent portfolios to assess firms’ R&D priorities [42]. Furthermore, AI-related patents are heavily concentrated in certain classification categories, most notably, CPC code G06F, which pertains to digital data processing [20].

Across both scientific and technological domains, several overarching trends in AI have emerged. The field is becoming increasingly interdisciplinary and is evolving from a focus on basic research to one centered on applied innovation. Core themes such as machine learning, deep learning, and neural networks consistently appear as central topics in both academic publications and patent documents [38,39]. While commonalities between AI-related scientific and technological trajectories have been acknowledged, there is a growing recognition of the need to integrate and interpret diverse data sources, rather than relying on a single dataset, in analyzing AI developments [3,4]. Nevertheless, comprehensive studies that systematically examine scientific and technological advancements in an integrated manner remain scarce. Only recently have scholars begun to explore the technological relevance of AI patents that cite scientific publications [18,35], or to investigate dual publication strategies among AI researchers who contribute to both academic literature and patent portfolios [43]. However, structured analyses that trace the transformation of specific scientific knowledge into technological innovation within the AI domain are still limited.

In addition, AI research and development continue to be concentrated in a small number of leading countries and institutions [38]. Despite this concentration, AI presents extensive opportunities for innovation in domains such as digital transformation, smart cities, and technology forecasting [44]. Many AI applications still rely heavily on foundational scientific theories [35], and numerous AI-related publications report significant scientific breakthroughs [45]. Moreover, corporations are increasingly engaging in academic publishing as a means to secure long-term technological leadership in AI [2]. In this context, identifying scientific research with high technological potential is essential for accelerating innovation and fostering more effective collaboration among academia, industry, and government stakeholders [18].

In response to these research gaps, the present study aims to analyze the structure and mechanisms of science-to-technology knowledge flow in the AI domain. Through this analysis, the study seeks to uncover the scientific foundations underlying AI technologies and generate insights to support strategic decision-making and policy development in an era of AI-driven innovation.

## 3. Methodology

### 3.1. Data

#### 3.1.1. AI patents.

To examine the temporal evolution of AI technologies, this study collected patent application data disclosed by the United States Patent and Trademark Office (USPTO) over a 20-year period from 2002 to 2021. The dataset was sourced from the Spring 2024 edition of the PATSTAT database.

AI-related patents were identified using a hybrid search strategy developed in prior studies, most notably the WIPO Technology Trends 2019 – Artificial Intelligence report [46]. This approach combines International Patent Classification (IPC) and Cooperative Patent Classification (CPC) codes with AI-relevant keywords, addressing the limitations of retrospective classification systems that often lag behind the emergence of new technologies. Compared to other emerging yet less standardized methods, such as unsupervised learning techniques or custom classification schemes [47], this hybrid strategy is widely recognized for its credibility and has been extensively adopted in the literature [48–50]. Developed by the World Intellectual Property Organization (WIPO), the strategy classifies AI patents across three dimensions: AI application fields, AI functional applications, and AI techniques.

This classification approach was rigorously validated through expert consultation, detailed exploration of classification systems, and benchmarking against existing AI patent landscape studies. In this study, we applied keyword-based criteria to the titles and abstracts of patents. To enhance the precision of SQL-based queries, we adapted WIPO’s methodology and referenced the implementation by Zhu and Motohashi [49], addressing the limitations of traditional keyword-based search expressions. It is important to note that the WIPO (2019) [46] search strategy required updates due to deletions and revisions in the IPC and CPC classification systems since its publication. To ensure classification accuracy, we incorporated all relevant changes by consulting the WIPO Deleted Symbols List and the Consolidated Revision Concordance List. The complete search query used for AI patent identification is provided in S1 Appendix in S1 File.

Applying this updated search strategy, we identified a total of 321,683 AI-related patents. To analyze the temporal dynamics of AI technological development, the dataset was segmented into four five-year periods: 25,246 patents were identified in Period 1 (2002–2006), 39,181 in Period 2 (2007–2011), 85,543 in Period 3 (2012–2016), and 171,713 in Period 4 (2017–2021).

#### 3.1.2. Scientific publications cited by AI patents.

To investigate the knowledge flow from science to AI technologies, this study compiled a dataset of scientific publications cited by the identified AI patents. These publications were retrieved from the Web of Science (WoS) database, which offers comprehensive bibliometric data on scholarly outputs. Using citation information from PATSTAT, we matched the references cited in AI patents to corresponding entries in the WoS database, resulting in a final dataset comprising 90,481 scientific publications. Although prior studies have noted that differences between citations added by applicants and those added by examiners may influence the interpretation of citation-based analyses [51,52], in our dataset only 182 examiner-added citations were identified among all patent–paper citation records, indicating that their impact is unlikely to be substantial; therefore, this distinction was not considered in the present analysis.

As BERTopic modeling requires textual content, only publications with available abstracts were included in the analysis. The temporal distribution of cited publications, including those cited by multiple patents, was as follows: 9,459 in Period 1 (2002–2006), 20,418 in Period 2 (2007–2011), 35,329 in Period 3 (2012–2016), and 28,625 in Period 4 (2017–2021).

The proportion of AI patents citing at least one scientific publication increased steadily from approximately 13% in Period 1–22% in Period 3. However, this share dropped sharply to 6% in Period 4. A closer inspection of Period 4 reveals that citations of scientific literature continued to rise between 2017 and 2019 but declined markedly from 2020 onward. This decline is likely attributable not to a weakening of science–technology linkages, but to inherent citation delays, arising from the time lag between patent application, publication, citation recording, and eventual inclusion in the PATSTAT database.

### 3.2. Methodology

#### 3.2.1. Research framework.

To analyze AI technology identification and the knowledge flow from science to technology, this study developed an integrated framework using AI patents and their cited scientific publications across four time periods. First, major Cooperative Patent Classification (CPC) main groups were extracted from AI patents to build co-occurrence networks. Weighted degree and betweenness centrality were then calculated to identify core and brokerage technologies, respectively, positioning them along a two-dimensional coordinate system that yielded four technology categories.

For each category, BERTopic modeling was applied to extract key topic clusters and representative keywords, and generative AI was used to assign concise, descriptive labels to enhance interpretability. The same procedures were applied to the scientific publications cited by patents in each category, capturing the thematic structure of the underlying scientific knowledge. By aligning patent and publication topics across categories and time periods, the framework systematically revealed how scientific knowledge evolves into technological innovation.

**Fig 1:**
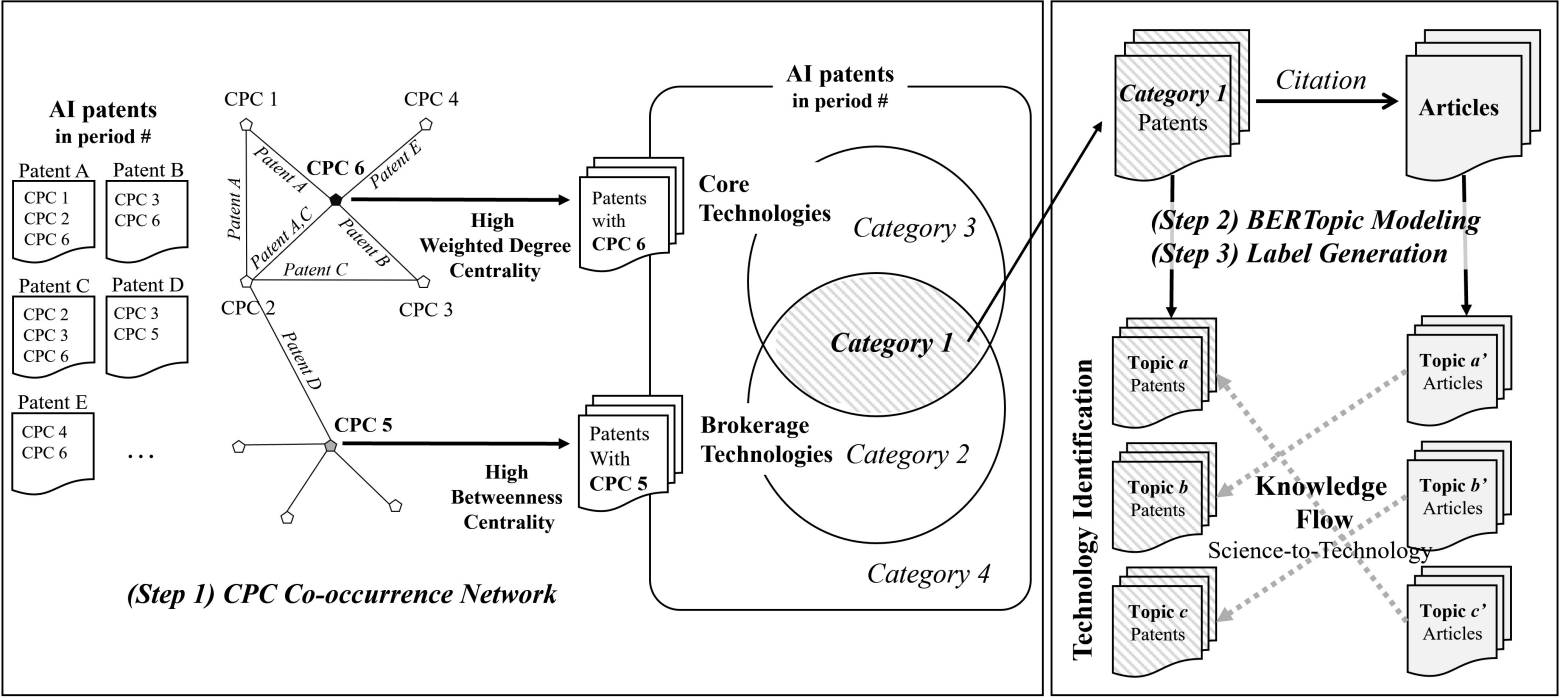
Research framework. This figure presents the overall research framework used to identify AI technologies and examine science-to-technology knowledge flow across four five-year periods spanning 2002 to 2021. The framework combines CPC-based network analysis for classifying core and brokerage technologies, BERTopic modeling for extracting topic clusters from both technological patents and their cited scientific publications, and generative-AI-based topic labeling to enhance interpretability. By aligning patent topics with those of the scientific literature they reference, the framework traces how scientific knowledge develops and transforms into technological knowledge within the AI domain.

The overall research framework, illustrated in Fig 1, visualizes these analytical steps and their interconnections, providing a comprehensive overview of how network analysis, topic modeling, and generative AI were integrated to trace the evolution of science-based technological development in the AI domain.

#### 3.2.2. CPC co-occurrence network analysis.

Artificial Intelligence (AI) exemplifies an emerging technology marked by rapid growth and intricate convergence across diverse fields, which has made the criteria for identifying specific technologies increasingly ambiguous [53]. In such a dynamic and complex technological landscape, it becomes essential to distinguish between core technologies, which serve as the foundation of the technological ecosystem, and brokerage technologies, which enable linkages and convergence among otherwise distinct domains [22,54].

Core technologies are defined as knowledge areas that occupy central positions within technological development. These technologies exhibit high levels of connectivity and co-citation with other technologies, making them valuable indicators for assessing the validity of technological trends [53,55,56]. In contrast, brokerage technologies mediate knowledge flow across disparate domains, acting as bridges that facilitate convergence. Their role provides critical insights into the economic validity and cross-domain impact of technologies [22,57,58]. Especially in multifaceted and interdisciplinary fields like AI, simultaneously considering both core and brokerage technologies enables more nuanced technology forecasting and foresight, traditional goals of technology identification research [59–61].

To classify AI technologies into these two categories, we conducted a co-occurrence network analysis based on the centrality of CPC codes. Using the compiled dataset of AI patents, separate co-occurrence networks were constructed for each of the four time periods. In each network, nodes represented main group CPC classifications and edges were established based on the co-occurrence of CPC codes within individual patents. The frequency of co-occurrence for each pair of CPCs served as the edge weight. To maintain a focus on technological classifications, CPC codes beginning with the letter “Y”, which denote non-technological or general tagging, were excluded from the analysis.

For each CPC main group, we calculated two key centrality measures: weighted degree centrality and betweenness centrality. Self-loops were removed from the network to better align with the objective of identifying inter-technology convergence. In addition, CPC codes that were directly used in the original search query were excluded from centrality rankings to avoid selection bias.

Weighted degree centrality reflects the total sum of edge weights connected to a node. A high weighted degree indicates that a CPC code frequently co-occurs with many others, signifying its importance as a core technology [54,57] within the AI patent landscape. Betweenness centrality, by contrast, quantifies how often a node lies on the shortest paths between other nodes [62]. A high betweenness centrality suggests that a CPC code functions as a brokerage technology [54,57], facilitating knowledge flow between otherwise unconnected technological domains [63]. Because edge weights in our network represent co-occurrence frequency, unlike in conventional betweenness calculations where weights often represent distance, we used the reciprocal of the edge weights when calculating betweenness centrality.

Following the construction of co-occurrence networks for each time period, we identified the top 10 main group CPCs based on weighted degree centrality and the top 10 based on betweenness centrality. We selected the top 10 CPC main groups because this threshold preserves the distinct characteristics of each technology while maintaining clear differentiation across categories. Using only the top five would result in categories that are too narrow, whereas expanding the threshold to the top 15–20 would lead to most patents in the sample being included across multiple groups, making category distinctions less meaningful due to excessive overlap. The top 10 captures roughly 70% of all patents, allowing for a balanced and appropriate level of representativeness without substantial duplication. Accordingly, patents containing CPC main groups ranked in the top 10 of either weighted degree centrality or betweenness centrality were extracted for subsequent analysis.

Each patent was then assigned to one of four technology categories: (1) Patents containing main group CPCs ranked in the top 10 for both weighted degree and betweenness centrality; (2) Patents containing CPCs ranked in the top 10 for betweenness centrality only (excluding those already included in category 1); (3) Patents containing CPCs ranked in the top 10 for weighted degree centrality only (excluding those already included in category 1); (4) Patents not falling into any of the above categories. This classification enabled a more granular understanding of the structural roles that different AI technologies play in the broader innovation landscape and provided the foundation for subsequent topic modeling and knowledge flow analysis.

#### 3.2.3. BERTopic modeling.

As described in the research framework, this study conducted two parallel topic modeling analyses, one on AI patents classified into four categories and the other on the scientific publications cited by those patents. Topic modeling plays a central role in identifying interpretable themes that represent the underlying knowledge structures in both technological and scientific domains. These generated topics form the basis for comparing how technology-oriented and science-oriented domains align within each of the four categories. Consequently, it is crucial to employ a topic modeling method capable of capturing the semantic richness of both patent and scientific texts.

To achieve this, we adopted BERTopic, a state-of-the-art topic modeling technique that leverages contextual embeddings to capture semantic similarity among documents. This enables the grouping of semantically similar texts and the extraction of interpretable keywords that represent each topic cluster [23]. Unlike traditional methods such as Bag-of-Words (BoW), which rely solely on word frequency, BERTopic incorporates semantic context, significantly enhancing both the coherence and interpretability of extracted topics, particularly important when dealing with complex and large-scale textual corpora such as patents and scientific articles [64].

In this study, BERTopic employed the pre-trained transformer-based language model “all-MiniLM-L6-v2” to generate contextual embeddings for each document. These embeddings were then reduced in dimensionality using Uniform Manifold Approximation and Projection (UMAP) to preserve their intrinsic semantic structure. Subsequently, Hierarchical Density-Based Spatial Clustering of Applications with Noise (HDBSCAN) was applied to group the documents into clusters. UMAP effectively condenses the embeddings while retaining meaningful relationships, and HDBSCAN enables the identification of semantically coherent clusters across documents. For each cluster, we applied class-based Term Frequency–Inverse Document Frequency (c-TF-IDF) to calculate the importance of terms while filtering out high-frequency but low-information words, thereby improving the representativeness of each topic. The top 10 keywords with the highest c-TF-IDF scores were selected to define each topic.

All analyses were conducted using the official BERTopic implementation developed by Grootendorst [59], publicly available on GitHub. As with other unsupervised learning approaches, careful tuning of BERTopic’s hyperparameters was essential to minimize unclustered or outlier documents. Specifically, the min_topic_size parameter was adjusted to fall between 1% and 3% of the total number of patents within each category. For instance, in a category with approximately 1,000 patents, the minimum topic size was set to 1%, ensuring that each valid topic cluster comprised at least 10 documents. To maintain topic interpretability, we also capped the number of topics (nr_topics_options) at 10 per category. Preprocessing steps included lowercasing text, removing special characters, and eliminating standard English stopwords such as prepositions, articles, and auxiliary verbs, all aimed at enhancing analytical precision.

This procedure was applied consistently across all datasets. For each of the four time periods, four patent categories were constructed, and the scientific publications cited by patents in each category were also collected. As a result, we conducted a total of 32 BERTopic analyses, 16 for the patent datasets (4 periods × 4 categories) and 16 for the scientific publication datasets (4 periods × 4 categories). This systematic approach ensured methodological consistency and enabled direct comparability between the technological and scientific domains, allowing us to extract topic structures and trace the patterns of knowledge flow from science to technology.

#### 3.2.4. Label generation.

While the keywords extracted from each BERTopic-derived cluster were generally appropriate for representing the underlying topics in both patent and publication analyses, the individual terms often lacked the intuitive clarity needed to infer the corresponding technological or scientific domains. To address this limitation, we employed generative AI to generate concise and contextually meaningful topic labels based on the extracted keywords. This approach enabled the semantic refinement of each cluster into an interpretable label, thereby enhancing the clarity and accuracy of topic interpretation across both technological and scientific contexts.

**Fig 2:**
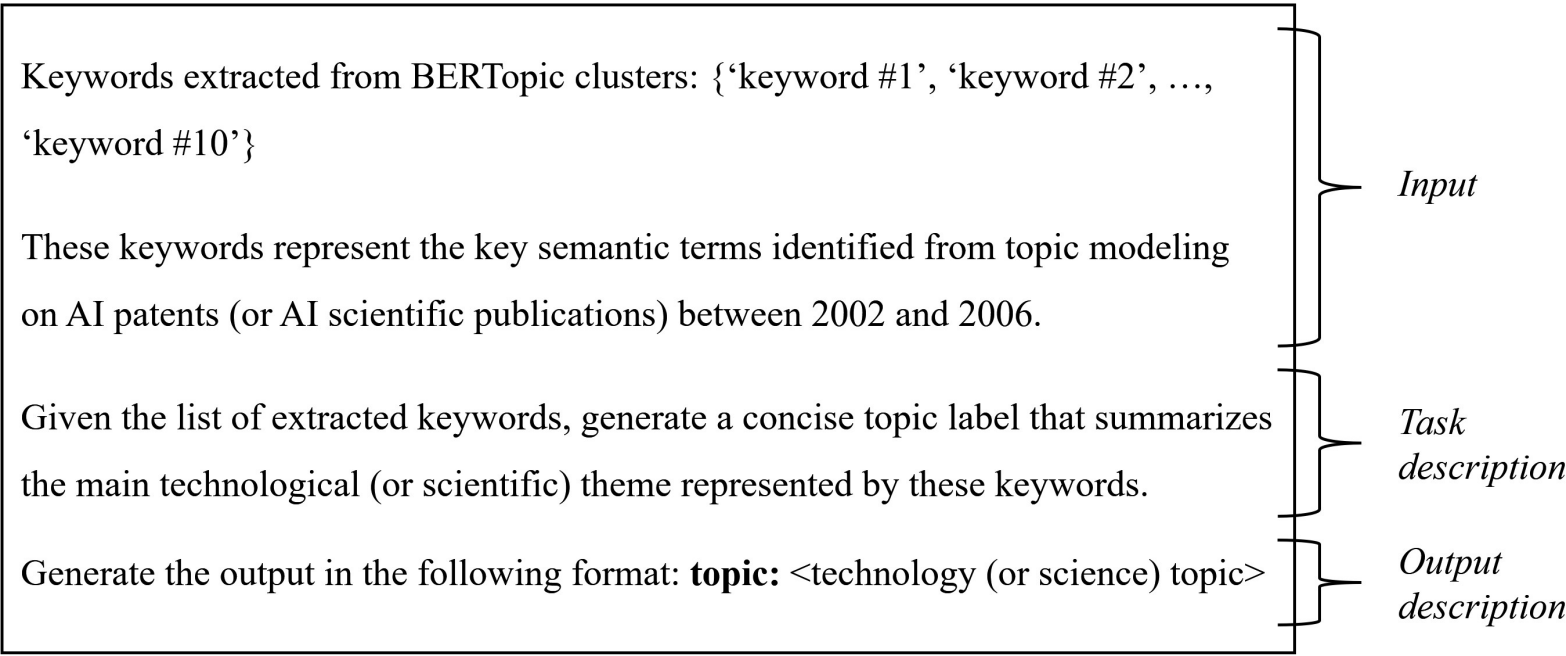
Designed prompt structure. This figure illustrates the structured prompt design used to generate interpretable topic labels for BERTopic clusters derived from both patents and scientific publications. The prompt is composed of an input section containing extracted keywords, a task description outlining the labeling objective, and an output instruction specifying the required format and constraints. This structured approach guides the generative AI model toward producing clear, relevant, and consistent topic labels across all clusters.

The labeling process was carried out using OpenAI’s ChatGPT-4o model and was applied consistently across both patent-based and publication-based topic clusters. This ensured methodological consistency between the two datasets and facilitated a coherent comparison of knowledge structures between science and technology. To further improve the coherence and domain relevance of the generated labels, we implemented a structured prompt design. Each prompt consisted of three distinct components, input, task description, and output instruction, which collectively defined the scope, expectations, and format of the model’s response. This structured prompting approach guided the language model toward producing interpretable, domain-appropriate labels. The full structure of the prompt is illustrated in Fig 2.

## 4. Results

### 4.1. Network analysis

To identify core and brokerage technologies within the AI domain across different time periods, we extracted the top 10 main group-level CPC codes based on two network centrality measures: weighted degree centrality and betweenness centrality. The results of this network analysis are summarized in Table 1.

**Table 1:** Top 10 main group CPCs by centrality in network analysis (5-year periods). This table presents the top ten main-group CPC codes for each five-year period of AI patent filings, identified through analysis of the CPC co-occurrence network and ranked according to weighted degree centrality and betweenness centrality. Weighted degree centrality captures the overall connectivity of a CPC with other CPCs and serves as an indicator of core technologies, while betweenness centrality captures the brokerage technologies that link otherwise heterogeneous technological areas.

Rank	Period 1(2002–2007)		Period 2(2008–2011)		Period 3(2012–2016)		Period 4(2017–2021)	
	weighted degree centrality	betweenness centrality	weighted degree centrality	betweenness centrality	weighted degree centrality	betweenness centrality	weighted degree centrality	betweenness centrality
1	G06F2221	G06F 16	G06F 3	G06F 16	G06F 3	G06F 3	G06N 20	G06N 20
2	G06F 3	G06F 3	G06F2221	G06F 3	G06F 16	G06F 16	G06F 3	G06F 3
3	H04N 1	G06F 18	G06F 16	G06F 1	G06F2203	G06V 20	G06F 16	G06V 20
4	G06F 1	G06F 1	G06F2203	G06Q 10	G06F 9	G06F 1	G06F 18	G16H 50
5	H04N 5	G06Q 10	G06F 9	G06V 20	H04L 67	G06F2203	G06V 20	G06F 1
6	H04L 9	G08G 1	G06F 1	G06F 18	G06F 12	G06N 20	G06V 10	G06F2203
7	G06F 16	G06V 20	G06F 12	G16B 40	G06F2221	G16H 50	G06F 9	G16B 40
8	H04N2201	G16B 40	H04N 1	G16H 50	H04L 63	G16B 40	H04L 67	G06F 16
9	H04L 63	G06F 12	G06F2212	G06N 20	G06F 1	G06F 18	G06Q 10	G06Q 10
10	H04N 23	G06V 10	H04L 63	G06F 12	G06F2212	G06V 40	G06F2203	G06F 18

An analysis of centrality measures revealed that CPC codes under the G06F classification (Electric Digital Data Processing) appeared most frequently across both weighted degree centrality and betweenness centrality, underscoring the foundational role of digital data processing methods, such as machine learning, deep learning, reasoning, and optimization, in AI technologies. Specifically, from Period 1 to Period 3, CPC codes related to data transformation (G06F 3) and database structures (G06F 16) consistently ranked among the top, indicating their central and enduring role within the AI technology ecosystem during these timeframes. In contrast, during the most recent period, CPC codes associated with machine learning (G06N 20) emerged as prominent, highlighting the shifting focus of AI development toward learning-based approaches.

Beyond these consistently top-ranked CPC codes, the remaining high-ranking codes varied between the two centrality measures, reflecting the diversity of technological components emphasized by each metric. Weighted degree centrality captures the volume of connections, while betweenness centrality identifies technologies that act as brokerages between otherwise disconnected areas, each offering a distinct perspective on technological significance within the AI landscape.

To refine and systematize the identification of core technologies, we applied BERTopic to a subset of patents containing main group CPCs that ranked highly in the centrality analysis. Based on their positions within the ecosystem, patents were classified into four categories. Category 1 includes patents containing main group CPCs that simultaneously ranked in the top 10 of both weighted degree and betweenness centrality, representing technologies that serve as both core and brokerage technologies. Category 2 consists of patents containing CPCs ranked in the top 10 of betweenness centrality, excluding those already in Category 1, and thus represents technologies that primarily serve as brokers connecting different technological areas. Category 3 comprises patents with CPCs ranked in the top 10 of weighted degree centrality, again excluding those in Category 1, representing core technologies with high connectivity but limited brokerage roles. Finally, Category 4 includes all remaining patents not classified into the previous three categories, representing peripheral or niche technologies within the broader AI domain.

### 4.2. BERTopic modeling & labeling

#### 4.2.1. AI technology identification.

As an example from Category 1 in Period 4, the BERTopic analysis clustered a total of 103,588 patents into four distinct topics, including one outlier cluster. The largest topic, comprising 61,995 patents, was characterized by the following ten keywords: [‘model’, ‘data’, ‘image’, ‘based’, ‘learning’, ‘method’, ‘vehicle’, ‘set’, ‘machine’, ‘user’]. Based on these keywords, a generative AI model labeled the topic as “Machine Learning Models for Intelligent Vision and Autonomous Systems.” Using the same procedure, topic keywords and corresponding labels were generated for all four time periods and across all four categories. The complete results are provided in S2 Appendix and S3 Appendix in S1 File. While some variation was observed across time periods and categories, the analysis revealed a notable consistency in the thematic characteristics of topic labels, regardless of time frame or centrality category.

**Fig 3:**
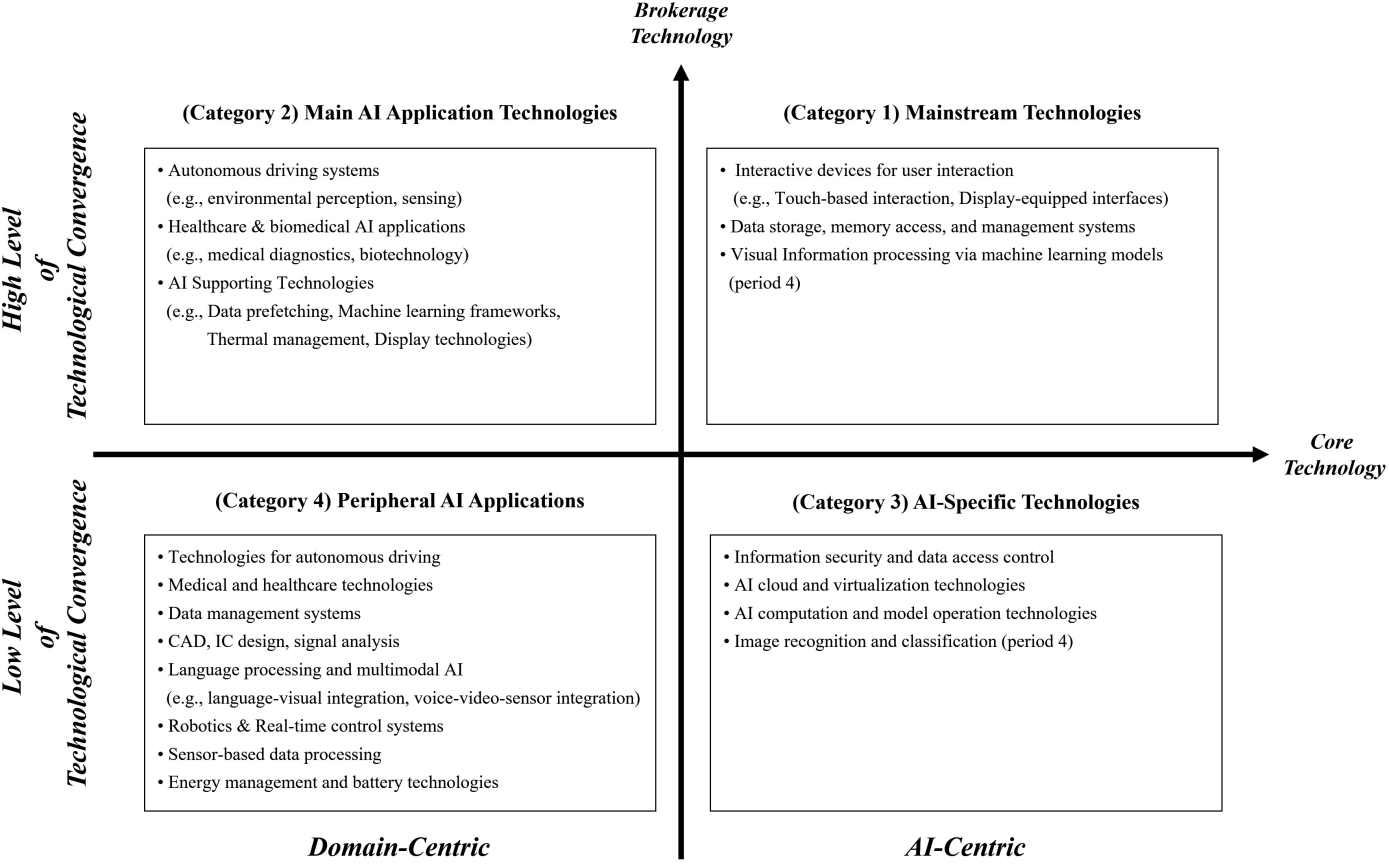
Technology Identification for AI Technologies. This figure visualizes major AI technology topics derived from centrality analysis of the CPC co-occurrence network. The horizontal axis indicates core-technology intensity (weighted degree centrality), ranging from domain-centric technologies on the left to AI-centric technologies on the right. The vertical axis represents brokerage (betweenness centrality), with higher positions showing stronger cross-domain connectivity. Based on these two dimensions, patents are grouped into four structural categories: mainstream, main AI application, AI-specific, and peripheral application technologies. Topic labels were generated using BERTopic and refined with generative AI. The figure summarizes consistent structural patterns across four five-year periods of AI patent data over 20 years.

The results are visualized in Fig 3, which positions the identified technologies along two axes based on their centrality characteristics. The horizontal axis represents whether a technology includes CPC codes ranked among the top in weighted degree centrality, indicating its centrality or prominence within the AI technology domain. Technologies located on the right-hand side are interpreted as AI-centric technologies, applicable across multiple areas of the AI ecosystem. In contrast, those on the left-hand side are classified as domain-centric technologies, tailored to specific application areas and less transferable across domains.

The vertical axis captures whether a technology includes CPC codes ranked among the top in betweenness centrality, reflecting its brokerage role in connecting otherwise distinct technological fields within AI. Technologies positioned higher on this axis are considered to have strong convergence potential, capable of facilitating interdisciplinary integration and demonstrating greater commercial scalability. Those positioned lower are generally more limited in their integrative function and reflect areas where AI application and industrial adoption are still emerging. As shown in Fig 3, the main topics identified through this classification are organized by time period and category, enabling a comparative view of how technological focus and roles have evolved.

Category 1 technologies, located in the first quadrant, are classified as “mainstream technologies” that simultaneously play both core and brokerage roles within the AI ecosystem. From Period 1 to Period 3, these technologies were largely associated with interface systems, enabling interaction between users and AI systems via devices equipped with displays. This reflects the foundational function of AI technologies: receiving user input, processing it, and returning output. In Period 4, there was a noticeable shift toward machine learning-based systems for autonomous visual processing, indicating an evolution toward more autonomous, perception-driven applications. Additionally, technologies related to data processing and memory management, which are essential to the operation of AI systems, consistently appeared among the most prominent technologies in this category across all periods.

Category 2 technologies, located in the second quadrant, are identified as “main AI application technologies” that primarily fulfill brokerage roles by enabling the convergence of diverse AI technologies. Throughout Periods 1–3, a dominant theme was the development of autonomous driving technologies, particularly those focused on environmental sensing and perception. This trend illustrates AI’s active deployment in real-world systems. Across all periods, applications in medical diagnostics, healthcare, and biotechnology were also prominent, with a significant increase in Period 4, suggesting rapid expansion of AI into these sectors. In addition, supporting technologies such as data prefetching, machine learning frameworks, thermal management for electronic components, and display systems emerged as notable technologies within this category.

Category 3 technologies, positioned in the fourth quadrant, represent “AI-specific technologies” that serve primarily core roles within the AI domain. These include infrastructure-level technologies that support AI implementation and those that enhance the development, operation, and performance of AI models. From Period 1 to Period 3, typical technologies included information security, data access control, AI computational techniques, and virtualization technologies for operating AI cloud platforms. In Period 4, the focus shifted to technologies related to resource allocation for cloud and edge AI systems, image data recognition and classification, and AI computing architectures optimized for enhanced model performance.

Category 4 technologies, located in the third quadrant, are categorized as “peripheral AI applications” and typically reflect emerging, niche, or experimental technologies. These efforts aim to apply AI across a wide range of specialized domains. In Period 1, a diverse set of technologies was observed, including autonomous driving, medical devices, data management, computer-aided design (CAD), integrated circuit (IC) design, signal analysis, language processing, and robotics. Period 2 featured early-stage technologies such as language-vision integration, real-time control, and evolving autonomous systems. In Period 3, advanced AI technologies became more prevalent, including sensor-AI integration, multimodal processing (voice, video, sensor data), battery technologies, and energy management. Notably, in Period 4, a majority of patents were related to sensor-based data processing and autonomous driving, reflecting the recent acceleration of AI adoption in mobility and sensing domains.

#### 4.2.2. Knowledge flow from science to technology in AI technology.

To investigate how scientific knowledge contributes to the development of AI technologies, we conducted BERTopic modeling and topic label generation on the scientific publications cited by AI patents. This analysis was performed across different time periods and technology categories. The resulting scientific topics were then compared with the previously identified technological topics to uncover patterns of knowledge flow from science to technology.

For instance, in Period 4, patents classified under Category 1 cited a total of 15,118 scientific publications. BERTopic modeling grouped these publications into five distinct topic clusters, one of which was identified as an outlier. The largest cluster comprised 11,294 publications and was characterized by the following keywords: [‘thyroid’, ‘ptc’, ‘quantum’, ‘follicular’, ‘papillary’, ‘data’, ‘saliency’, ‘using’, ‘results’, ‘patients’]. Based on these keywords, the cluster was labeled “Deep Learning Models for Image and Network Analysis.”

This scientific topic closely aligned with the corresponding technological topic for Category 1, labeled “Machine Learning Models for Intelligent Vision and Autonomous Systems.” The strong semantic connection between these topics illustrates a clear case of knowledge transfer, where scientific research provides the conceptual foundation for technological innovation. Detailed BERTopic clustering results and the full list of topic labels for each category and time period are presented in Appendices S2 and S3.

**Fig 4:**
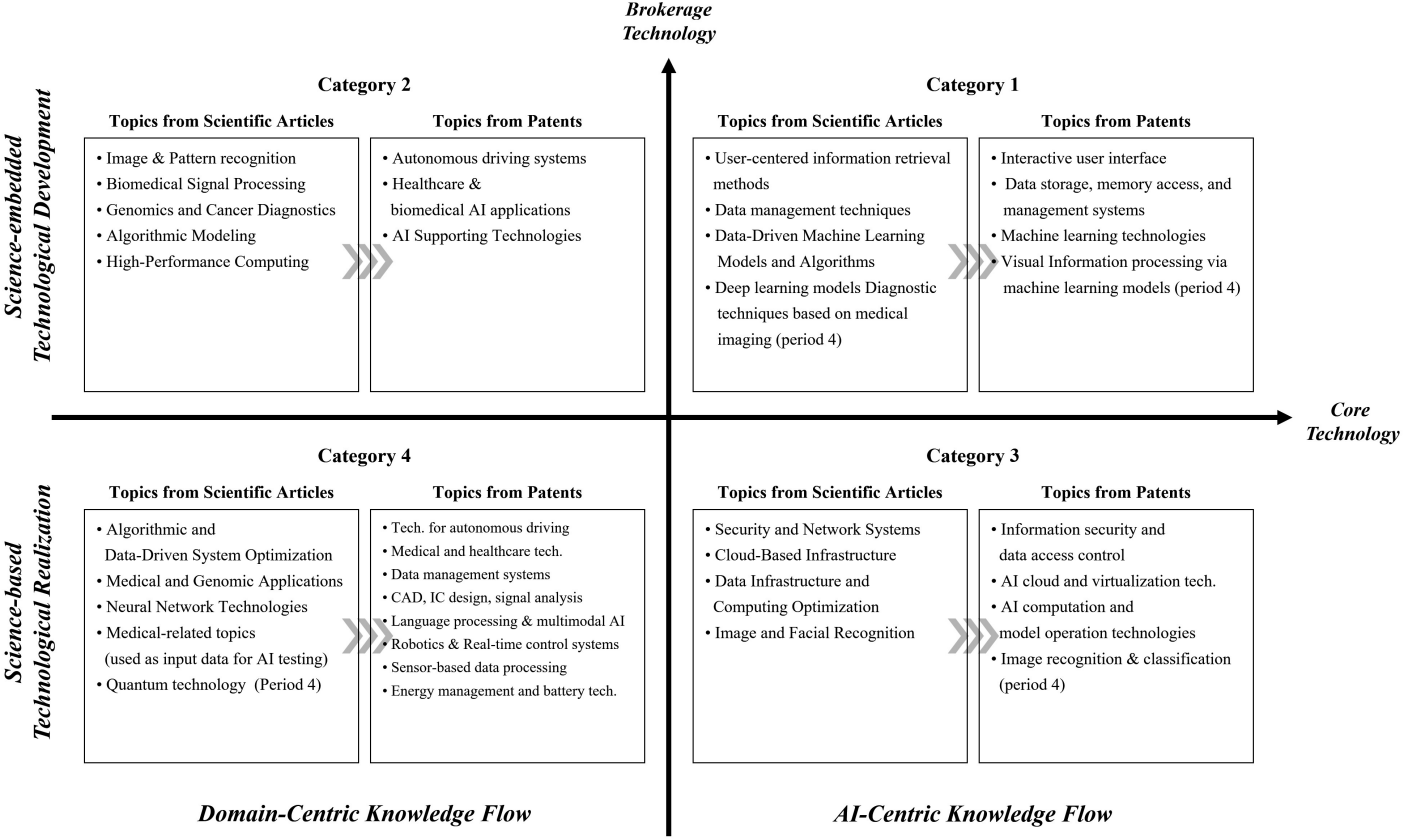
Knowledge Flow from science to technology in AI Technologies. This figure compares the semantic alignment between technological topics extracted from AI patents and scientific topics from their cited publications, highlighting category-specific patterns of science-to-technology knowledge flow. The horizontal axis represents the degree of core technological orientation, ranging from domain-centric flows on the left to AI-centric flows on the right. The vertical axis distinguishes two modes of scientific utilization: Science-embedded Technological Development at the top, where technological objectives guide the embedding and adaptation of scientific knowledge, and Science-based Technological Realization at the bottom, where scientific findings are directly translated into technological solutions. The four resulting quadrants capture the intersections of these dimensions and reveal distinct structural patterns of knowledge flow. The figure synthesizes stable patterns observed across four consecutive five-year periods covering 20 years of AI patent data.

Drawing on this analysis, we examined the semantic relationships between technological topics and their cited scientific counterparts across all categories and time periods. This allowed us to identify distinct temporal and structural patterns of knowledge flow from science to technology, which are visually summarized in Fig 4.

In Fig 4, the horizontal axis represents the degree of core technological centrality, capturing how central a topic is within the broader AI technology landscape. Topics located on the right-hand side are characterized as AI-centric scientific knowledge, directly supporting the advancement of core AI technologies. In contrast, topics on the left-hand side reflect domain-centric knowledge flows, where scientific knowledge is applied to specific industries or application areas. Overall, AI-centric topics tend to exhibit higher semantic alignment between scientific and technological domains, indicating a more direct and cohesive flow of knowledge and underscoring the foundational role of scientific research in the development of core AI capabilities.

The vertical axis represents the degree of brokerage, which distinguishes between two qualitatively different modes of science-to-technology knowledge transfer. Topics positioned in the upper region of the graph illustrate a “Science-embedded Technological Development” pattern. In this pattern, technological goals shape the scope and application of scientific knowledge, which serves as a critical conceptual resource enabling innovation. These topics typically involve leading AI technologies and their major application areas, highlighting the strategic integration of scientific insights into the development process.

In contrast, topics located in the lower region reflect a “Science-based Technological Realization” pattern. Here, the structure of the technological topics closely mirrors that of the cited scientific knowledge, suggesting that technological advances are driven by the direct implementation of existing scientific findings. Rather than using science to shape innovation trajectories, this pattern reflects a process in which scientific principles are directly translated into technological design, emphasizing realization over exploration.

Category 1 technologies (first quadrant) exhibit a clear pattern of AI-centric and science-embedded technological development. The scientific publications cited in this category primarily focus on user-centered information retrieval and data management techniques, core conceptual foundations for AI implementation, particularly in areas such as human–machine interaction. For example, in Period 4, patents centered on machine learning systems frequently referenced scientific work in deep learning, network analysis, and medical imaging diagnostics. These areas offer foundational knowledge essential for enabling AI technologies, indicating that Category 1 encompasses the core of AI development, where scientific research supports technological development.

Category 2 technologies (second quadrant) demonstrate a domain-centric, yet still science-embedded, pattern of development. These technologies are primarily situated in high-adoption fields such as autonomous driving, healthcare, and medical diagnostics, domains where AI applications are rapidly proliferating. The cited scientific literature in this category emphasizes topics like image recognition, pattern analysis, and biomarker identification for disease detection. While the scientific and technological topics may not match precisely, they are conceptually aligned, particularly around domain-specific challenges. Category 2 technologies prioritize real-world application over foundational AI theory, and thus rely on the active integration of scientific knowledge tailored to specific domains.

Category 3 technologies (fourth quadrant) reflect an AI-centric and science-based technological realization pattern. In this quadrant, there is a strong semantic alignment between scientific and technological topics, particularly around systemic or service-level functions. Key themes on the technological side include information security, data access control, and AI cloud platform operations. These are closely mirrored by corresponding scientific topics such as cybersecurity, memory management, and cloud computing. Rather than shaping new innovations, scientific knowledge in this context is directly implemented, suggesting that Category 3 technologies represent the practical application of established scientific theories and principles derived from foundational AI research.

Category 4 technologies (third quadrant) are characterized by peripheral AI applications and a domain-centric knowledge flow. In some cases, scientific and technological topics show clear alignment, indicating a science-based technological realization pattern. Fields such as data management, medical technologies, and battery systems demonstrate topical overlap between patents and the scientific literature. Interestingly, a number of scientific topics, particularly in the medical field, did not explicitly match the patent topics. This may reflect the practical reality that high-quality medical datasets, while not the focus of patented innovations, are vital for training and validating AI models. Additionally, in Period 4, topics related to quantum technology emerged in the cited scientific literature, highlighting this framework’s ability to capture early-stage, experimental developments in the AI landscape. Overall, Category 4 represents a more exploratory and interdisciplinary space, where the applicability of emerging scientific knowledge is still being tested and refined.

Collectively, these findings show that the knowledge flow from science to technology in AI is neither uniform nor linear. Instead, it is multifaceted and structurally differentiated, shaped by two key dimensions: (1) whether the technology is AI-centric or domain-centric, and (2) whether scientific knowledge is embedded within technological development or serves as the basis for direct technological realization. The evolution of AI technologies thus lies along a continuum, ranging from the functional and applied integration of scientific knowledge to its principle-based realization. The interaction of these two dimensions offers valuable insight into how scientific foundations shape, support, and steer the formation and diffusion of AI innovation.

### 4.3. Additional analysis

#### 4.3.1. Robustness check.

The main analysis divided the 2002–2021 period into four five-year intervals to classify technologies and identify patterns in science-to-technology knowledge flow. To test the robustness of these findings and examine whether the identified patterns were specific to five-year intervals, we conducted an additional analysis using two broader timeframes, splitting the data into two ten-year periods: the first (2002–2011) and second half (2012–2021). The same methodology was applied, including network analysis based on the CPC main groups of AI patents, centrality-based categorization of technologies, and linkage analysis with the scientific publications cited by those patents.

The results of the network analysis and the top-ranked CPC main groups for each decade are presented in Table 2. Notably, no entirely new CPC main groups emerged beyond those identified in the five-year analysis. In both decades, the G06F category (Electric Digital Data Processing) remained dominant. However, a key distinction in the second decade was the rise of G06N 20 (machine learning), which ranked highly in both weighted degree and betweenness centrality, signaling a notable shift in the computational foundations of AI technologies. Compared to the first decade, which emphasized data storage, structures, and communication technologies, the second decade marked a transition toward more algorithm-intensive and perception-driven areas, including machine learning, information processing algorithms, and visual data processing. This shift was also reflected in increased centrality of domains like medical information systems and user interfaces, pointing to a broader diversification in AI applications over time.

**Table 2:** Top 10 main group CPCs by centrality in network analysis (10-year periods). This table presents the top ten main-group CPC codes for each ten-year period of AI patent filings, identified through CPC co-occurrence network analysis and ranked by weighted degree centrality and betweenness centrality. Weighted degree centrality reflects the extent to which a CPC connects to other CPCs and indicates its role as a core technology, while betweenness centrality captures its brokerage technology in bridging distinct technological domains.

Rank	2002 - 2011		2012-2021	
	weighted degree centrality	betweenness centrality	weighted degree centrality	betweenness centrality
1	G06F2221	G06F 16	G06F 3	G06F 3
2	G06F 3	G06F 3	G06N 20	G06N 20
3	G06F 16	G06F 18	G06F 16	G06F 16
4	G06F 1	G06Q 10	G06F 18	G06V 20
5	G06F2203	G06F 1	G06F 9	G06F 1
6	G06F 9	G08G 1	G06V 20	G16H 50
7	H04N 1	G06V 20	G06V 10	G06F2203
8	H04L 63	G16B 40	H04L 67	G16B 40
9	H04N2201	G06N 20	G06F2203	G06F 18
10	G06F 12	G06F 12	G06Q 10	G06Q 10

When examining betweenness centrality, the first decade was dominated by data storage and processing groups such as G06F 16, G06F 3, and G06F 18. By the second decade, the central roles shifted toward machine learning (G06N 20) and visual information processing (G06V), along with the emergence of new application domains such as medical information systems (G16H 50) and user interfaces (G06F 2203), indicating an expansion in the range of AI-related technologies. Despite these differences, the overall structure and dominant groups remained largely consistent with the results of the five-year interval analysis.

**Fig 5:**
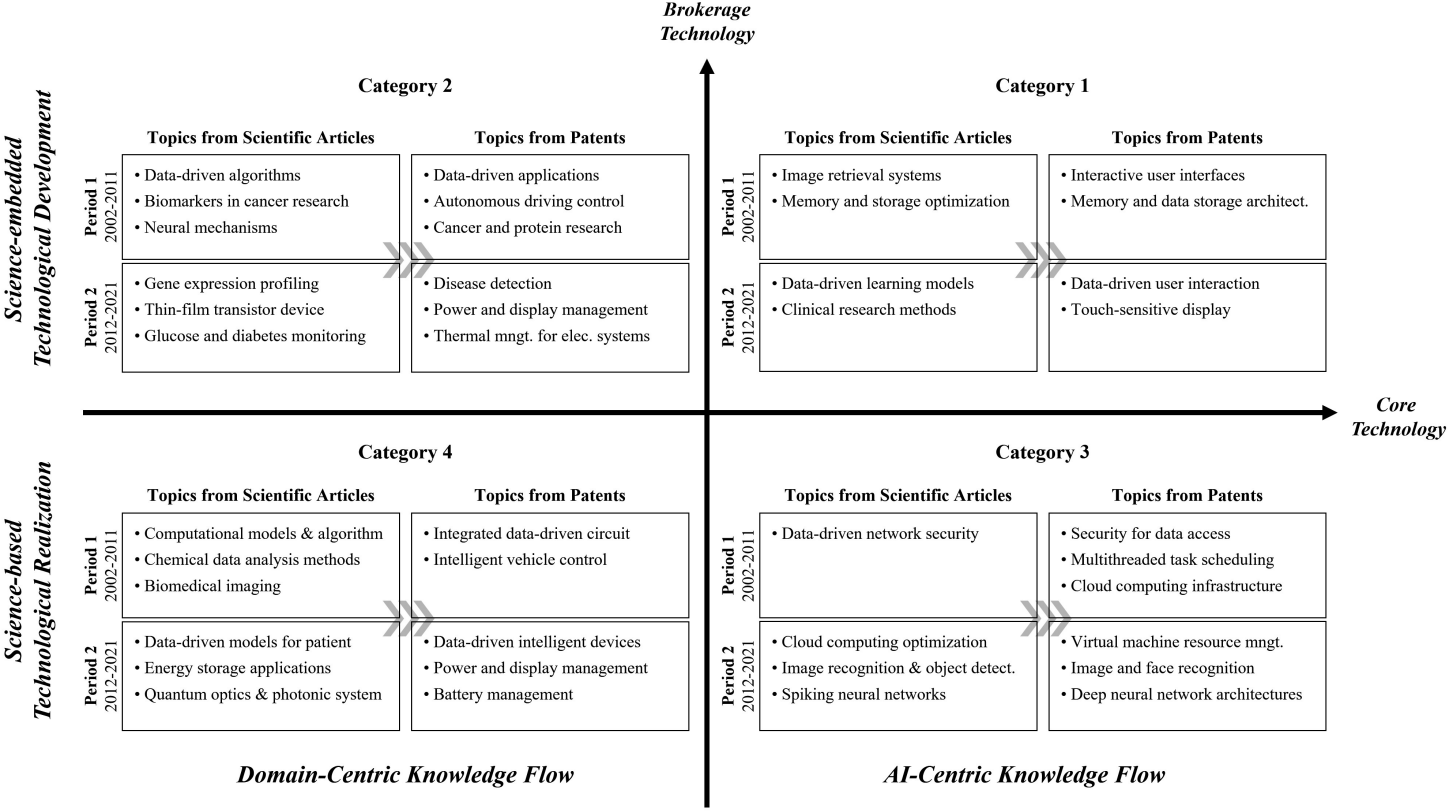
Knowledge Flow from science to technology in AI Technologies (10-years periods). Unlike the earlier five-year analyses, this figure presents a ten-year aggregated analysis to assess the robustness of science-to-technology knowledge flow patterns. Each category is divided into two stacked panels, with the upper and lower boxes representing the first and second ten-year periods, respectively. As before, the horizontal axis reflects core technological centrality, and the vertical axis captures brokerage-based modes of knowledge integration. The results show that core technologies maintain AI-centric knowledge flows, while brokerage technologies exhibit science-embedded patterns in which technological objectives guide the embedding and use of scientific knowledge. The figure confirms that these structural patterns remain stable even when the temporal aggregation window is expanded.

Building on this, we extracted the top CPC main groups and applied the same category-based topic modeling to both patents and their cited scientific publications. The results, visualized in Fig 5., reveal that the knowledge flow patterns remained largely consistent with those identified in the five-year analysis.

Along the horizontal axis, technologies classified as core tended to center around AI-centric domains such as data processing, cybersecurity, cloud computing, and virtual environments, areas fundamental to the development and deployment of AI systems. The cited scientific topics in these categories were also closely aligned with these technological domains, reflecting a strong AI-centric knowledge flow.

In contrast, technologies with high brokerage values were more strongly associated with application-driven domains, including user interaction, autonomous driving, and healthcare. Here, the scientific knowledge tended to provide conceptual and methodological foundations to support implementation, corresponding to a Science-embedded Technological Development pattern. At the opposite end of the brokerage axis, we observed a stronger one-to-one alignment between technological and scientific topics, indicating a Science-based Technological Realization pattern in which scientific principles are more directly translated into technological applications. These patterns are visually summarized in Fig 5, which illustrates the knowledge flow from science to technology in AI technologies across 10-year periods. The full results of BERTopic clustering, including extracted keywords and generated topic labels for each category and time frame, are provided in S4 Appendix and S5 Appendix in S1 File.

#### 4.3.2. Case study.

To assess whether the typological patterns observed through topic modeling are reflected in real-world examples, we conducted a case study of individual patents and their cited scientific publications. For each category in Period 4 (2017–2021), we randomly selected one representative patent along with one of its cited scientific articles. The full texts of these documents are provided in Appendix S6 in S1 File.

The patent sampled from Category 1 reflects a mainstream AI technology that combines both core and brokerage characteristics, aligning with an AI-centric and science-embedded technological development pattern. The patent, filed in 2019 under the topic “Machine Learning Models for Intelligent Vision and Autonomous Systems,” describes a deep learning-based automated image analysis system that tracks particle motion in video microscopy. One of its cited papers, published in 2009, details experimental and theoretical methods for tracking particles in living cells using imaging techniques. This case exemplifies how foundational scientific knowledge, both physical and biological, has been embedded in the implementation of a core AI technology, capturing the essence of Category 1.

The Category 2 patent represents application-oriented AI technologies with strong brokerage attributes, characterized by a domain-centric and science-embedded technological development flow. Filed in 2020 under the topic “AI-Based Medical Diagnosis and Patient Modeling,” the patent proposes an AI-driven cerebrovascular analysis system that detects vascular abnormalities from brain imaging data. The cited scientific article, published in 2006, presents mathematical and computational methods for analyzing vascular structures from medical images. This case illustrates the integration of fundamental scientific principles from the medical domain into a specialized AI application, embodying the defining traits of Category 2.

The example from Category 3 represents a technology that supports core AI functions and exhibits an AI-centric and science-based technological realization pattern. The selected patent, filed in 2017 under the topic “Virtualized Computing Services and Resource Allocation,” proposes a server security mechanism that mitigates denial-of-service (DoS) attacks by manipulating resource consumption. One of its cited references, a scientific publication from 2002, discusses theoretical security protocols for defending against DoS attacks. This alignment demonstrates the direct translation of scientific theory into technical implementation, reflecting the operationalization of scientific principles typical of Category 3.

In Category 4, the selected patent represents a peripheral AI application with relatively weak core and brokerage characteristics. It follows a domain-centric and science-based technological realization pattern. The 2017 patent, categorized under “AI-Integrated Vehicle Systems and Sensor-Based Data Processing,” introduces a deep learning model designed to predict an author’s personality traits based on text input. The cited scientific paper, published in 2016, explores AI modeling techniques for recognizing personality traits (Big Five) using the temporal patterns of conversational turn-taking. This case demonstrates how research from a peripheral domain, psychology, has been directly applied in the development of an AI-based predictive model, exemplifying the character of Category 4.

Taken together, the sampled patents and their associated scientific publications closely reflect the categorical distinctions identified through our broader analysis. The observed patterns of knowledge flow, from science to technology, are consistent with those derived through topic modeling. These case-level findings support the validity of the typology and confirm the real-world applicability of the proposed analytical framework.

#### 4.3.3. Regression analysis.

To complement the qualitative findings, we conducted a quantitative analysis at the patent level to examine how actively each technology category draws on scientific knowledge. Of the 321,683 AI patents initially identified, we excluded those with incomplete control variable data, resulting in a final sample of 319,597 patents.

The dependent variable was the number of scientific publications cited by each patent. The main independent variables were dummy indicators for Categories 1–4, and control variables included the number of claims(CLAIMS), patent family size(FAMILY), number of inventors(INVENTORS), number of applicants(APPLICANTS), and patent age(PAT AGE) (defined as years from the application year to 2021). Control variables were log-transformed to reduce skewness and scale differences. Descriptive statistics and the correlation matrix for all variables are provided in Appendix S7 in S1 File, and no high correlations were observed among the variables. Because the dependent variable is a count variable, and nearly 90% of patents cited no scientific publications, we applied a Zero-Inflated Negative Binomial (ZINB) regression model.

Table 3 presents the regression results. In our main specification (Model 2), we find that brokerage technologies are associated with significantly higher levels of scientific citation. This supports the idea that technologies situated in brokerage positions, often characterized by cross-domain integration and commercial versatility, draw more heavily on scientific inputs during development.

**Table 3:** ZINB regression results for scientific citation intensity by technology category. This table presents the Zero-Inflated Negative Binomial (ZINB) regression results examining the relationship between four technology categories, defined by their core and brokerage characteristics, and the number of scientific publications cited at the patent level. The dependent variable is the number of scientific publications cited by each patent, while the independent variables are dummy indicators for the four technology categories. The control variables include CLAIMS, FAMILY, INVENTORS, APPLICANTS, and PAT AGE, all of which were log-transformed to adjust for skewness and scale differences. Period dummies are incorporated in Model 3. The results indicate that Category 2 patents exhibit the highest level of scientific citation, followed by Categories 1, 3, and 4.

Dependent variable	Number of cited publications	Model 1	Model 2	Model 3
Control variables	CLAIMS	0.7110***	1.0463***	1.0675***
		(0.0131)	(0.0083)	(0.0105)
	FAMILY	0.2492***	0.2434***	0.2436***
		(0.0120)	(0.0116)	(0.0187)
	INVENTORS	0.4486***	0.4288***	0.3015***
		(0.0200)	(0.0194)	(0.0302)
	APPLICANTS	0.0462*	0.0429*	0.4323***
		(0.0264)	(0.0253)	(0.0405)
	PAT AGE	0.9606***	0.8507***	
		(0.0153)	(0.0147)	
	PERIOD 2			0.4264***
				(0.0545)
	PERIOD 3			0.5012***
				(0.0480)
	PERIOD 4			−0.2352***
				(0.0494)
Independent variables	CATEGORY 1		−0.4674***	−0.5349***
			(0.0197)	(0.0312)
	CATEGORY 2		0.8392***	0.8367***
			(0.0323)	(0.0455)
	CATEGORY 3		−0.8236***	−0.8737***
			(0.0273)	(0.0405)
CONSTANT		−2.8718***	−5.8247***	−4.6382***
		(0.098)	(0.0476)	(0.0741)
Observations		319,597	319,597	319,597
LnAlpha		2.621***	2.586***	2.619***
LR Chi2		7835.77***	27027.99***	15130.43***
Log Likelihood		−156967.4	−155954.6	−156938.8

Notes: * p < 0.1, ** p < 0.05, *** p < 0.01. Standard errors are in parentheses below the coefficients.

At the category level, Category 2 patents (domain-centric applications such as healthcare and autonomous systems) exhibited the highest number of scientific citations and were the only category showing a statistically significant positive effect. This suggests that AI applications in high-adoption domains actively incorporate established scientific methods and concepts to facilitate domain-specific implementation.

In contrast, Category 1 (mainstream AI technologies) and Category 3 (AI-specialized infrastructure technologies) both showed significant negative relationships with scientific citations. Although these categories represent foundational and technically advanced AI developments, they appear to rely less on direct citation of scientific literature. Notably, Category 3 displayed strong conceptual alignment with scientific knowledge in our earlier qualitative analysis, even though citation volumes were relatively low, implying that scientific principles are implemented directly but not necessarily extensively cited.

Category 1, despite representing central AI technologies, showed an even lower citation coefficient, possibly reflecting greater dependence on experimental or theoretical knowledge rather than frequent referencing of published science. Category 4 patents showed the lowest citation activity, as indicated by the significantly negative coefficient for the constant term. This aligns with their characterization as peripheral technologies, where scientific references are less frequently utilized.

Among control variables, patent age was positively associated with citation count, meaning newer patents cited fewer scientific publications. However, when we replaced patent age with period dummies in Model 3, Periods 2 and 3 showed higher citation counts compared to Period 1, while Period 4 was significantly negative. This decline is likely attributable to citation lag, a delay between patent filing, disclosure, and the indexing of citations in the dataset.

Overall, the findings indicate that AI applications in external domains make the most extensive use of scientific literature, reflecting an active and explicit process of knowledge transfer. In contrast, AI-native technologies, while conceptually rooted in science, tend to engage with scientific knowledge in a more implicit or embedded manner, suggesting distinct pathways of science–technology integration.

## 5. Discussions and implications

This study developed an integrated analytical framework to identify AI technologies and examine the knowledge flow between science and technology. By introducing a dual classification system, distinguishing between core technologies that drive the AI ecosystem and brokerage technologies that bridge disparate technological domains, the framework offers a semantic and structural understanding of how scientific knowledge supports AI innovation. Combining network analysis, BERTopic-based topic modeling, and generative AI–based labeling, we systematically clustered AI patents over four time periods and analyzed how science-to-technology knowledge flows unfold within each cluster.

The findings show that the way scientific knowledge is used in AI innovation varies depending on a technology’s structural role. Technologies performing a brokerage function tend to draw more heavily on conceptual insights from science, while the distinction between AI-centric and domain-centric applications aligns with whether a technology plays a core or peripheral role. Based on these two dimensions, the study identifies four distinct knowledge flow patterns, highlighting the layered and context-specific ways in which science contributes to technological development in AI. These insights carry significant implications for both firms and policymakers, particularly in light of AI’s rapid diffusion and shifting centers of technological leadership. For firms, understanding how technologies are situated along the core–brokerage spectrum, and how they connect to scientific knowledge, can inform strategic decisions around portfolio management and R&D investments.

Firms or regions focused on brokerage technologies, such as autonomous vehicles or diagnostic AI systems, are likely to benefit from closely engaging with scientific literature to integrate essential concepts into applied solutions. In contrast, organizations specializing in core technologies, such as cloud infrastructure, cybersecurity, or resource allocation, may need to focus on translating foundational scientific principles into robust, scalable technological systems. For mainstream AI technologies that serve both core and brokerage functions, such as user-facing intelligent devices or systems enabling real-time data processing, R&D efforts should balance scientific conceptual grounding with AI-driven technological enhancement.

From a managerial perspective, these findings underscore the importance of tailoring capability-building strategies to the nature of a firm’s technologies. Because the effectiveness of such strategies depends in part on absorptive capacity, the proposed framework helps firms assess their position in the knowledge ecosystem and align their R&D trajectories accordingly. Firms can use this framework to determine whether their existing technologies are core or brokerage, and to design appropriate roadmaps for integrating external scientific knowledge into internal innovation efforts.

From a policy standpoint, the results offer valuable guidance for shaping national or regional R&D strategies in AI. For economies aiming to close technological gaps, an effective approach may begin with support for brokerage technologies that leverage existing industrial strengths, followed by targeted investment in foundational core technologies. The structural understanding of science-to-technology flows presented in this study also supports the design of policies that bridge basic research and industrial innovation. In growing AI-centric technologies, closer integration with basic science is essential, while for domain-centric applications, policies should encourage the adaptation of existing scientific knowledge to real-world technological needs. These strategies can be further refined at the category level and inform initiatives such as targeted R&D funding or university–industry partnerships.

While this study offers an integrative perspective, several limitations remain. First, the framework focuses on science-to-technology flows, without accounting for other distinctive features of the AI knowledge ecosystem, such as preprint culture, open benchmarks, or rapid diffusion cycles, that may influence innovation dynamics. Moreover, reverse knowledge flows from patents to scientific literature could not be captured due to data limitations. Future research should explore these dimensions using complementary methodologies to develop a more comprehensive picture of AI innovation.

Second, the study finds that mainstream technologies in Category 1 are often centered on intelligent, interactive devices, a direction aligned with AI’s broader goal of human-centered integration. This trajectory, where AI technologies converge with human interaction, reflects a major trend in AI’s evolution (Raman et al., 2025). However, due to this study’s specific focus on science-to-technology flows, the broader implications of this shift were not explored in depth. Future work could extend the framework to engage with the ethical, social, and technical challenges surrounding developments like Artificial General Intelligence (AGI), offering a more multidimensional view of AI’s future.

Finally, because the framework was applied exclusively to AI, further research is needed to test its generalizability across other emerging technologies. Although BERTopic and generative AI represent state-of-the-art NLP tools, topic interpretation may still involve subjective elements due to prompt design and parameter tuning. These limitations also point to promising directions for future inquiry. Applying this framework to fields like biotechnology, quantum computing, or renewable energy could reveal whether the observed knowledge flow patterns are technology-specific or more widely applicable. Additionally, combining this approach with qualitative methods, such as interviews with R&D professionals or in-depth case studies, could provide richer contextual understanding and deepen the strategic and policy relevance of the findings.

## Supporting information

S1 Table Query strategies for AI patents. (DOCX)

S2 Fig Topic keywords from BERTopic modeling. (DOCX)

S3 Fig Topics from label generation. (DOCX)

S4 Fig Topic keywords from BERTopic modeling (10-years periods). (DOCX)

S5 Fig Topics from label generation (10-years periods). (DOCX)

S6 Fig Representative patents and their cited scientific publications by category (Period 4: 2017–2021). (DOCX)

S7 Table Descriptive statistics and correlations for additional regression analysis variables. (DOCX)

S1 File Appendix. (DOCX)
