# Supplementary figures and images for: Knowledge flows from science to AI technology: Identifying core and brokerage technological roles
Source: PLoS One. 2026 Feb 19;21(2):e0341005. doi: 10.1371/journal.pone.0341005 (PMC12919798; doi:10.1371/journal.pone.0341005)

# **S2 Figure. Topic keywords from BERTopic modeling**

**Period 1 (2002-2006)**

**
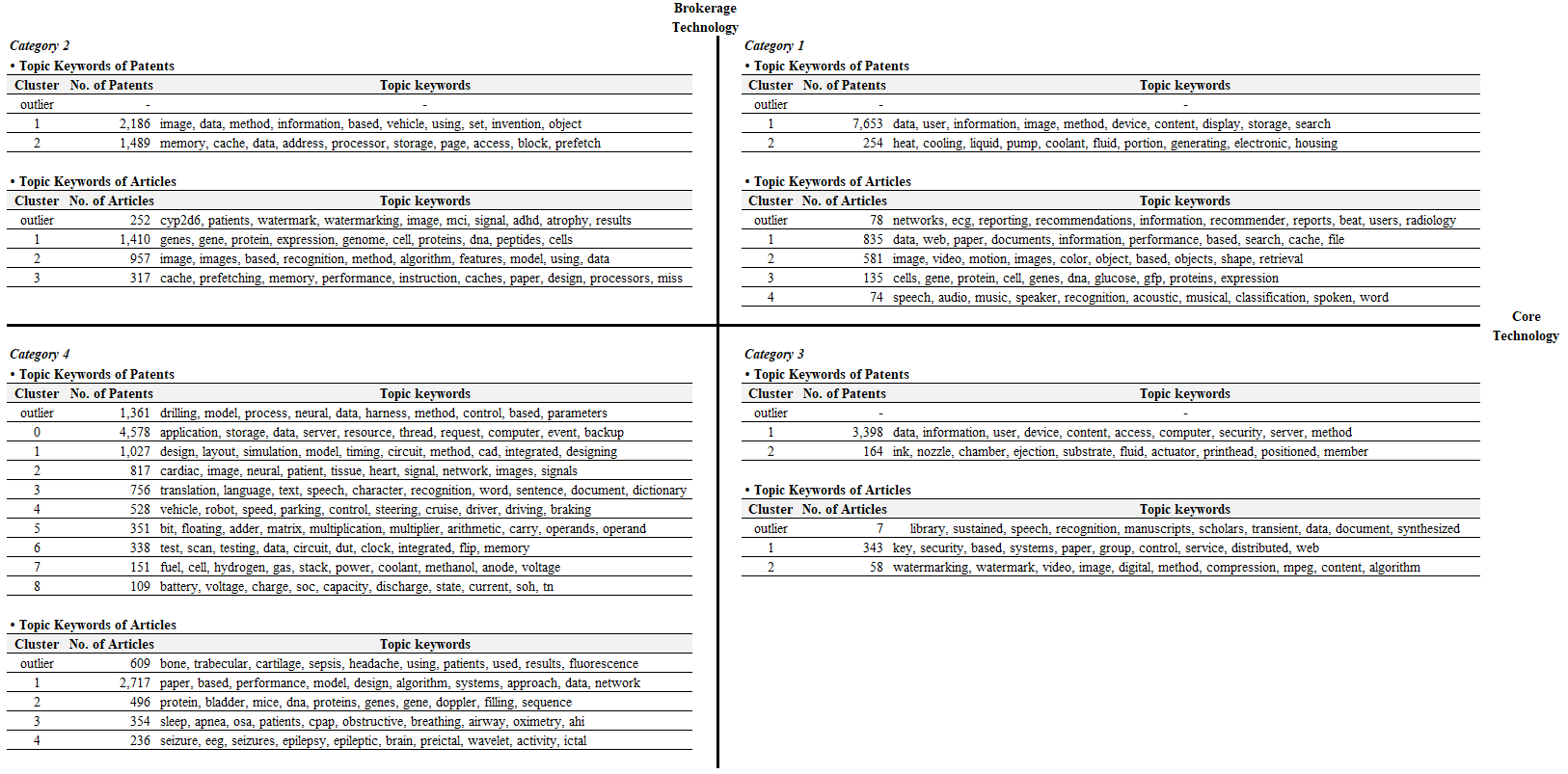
**

**Period 2 (2007-2011)**


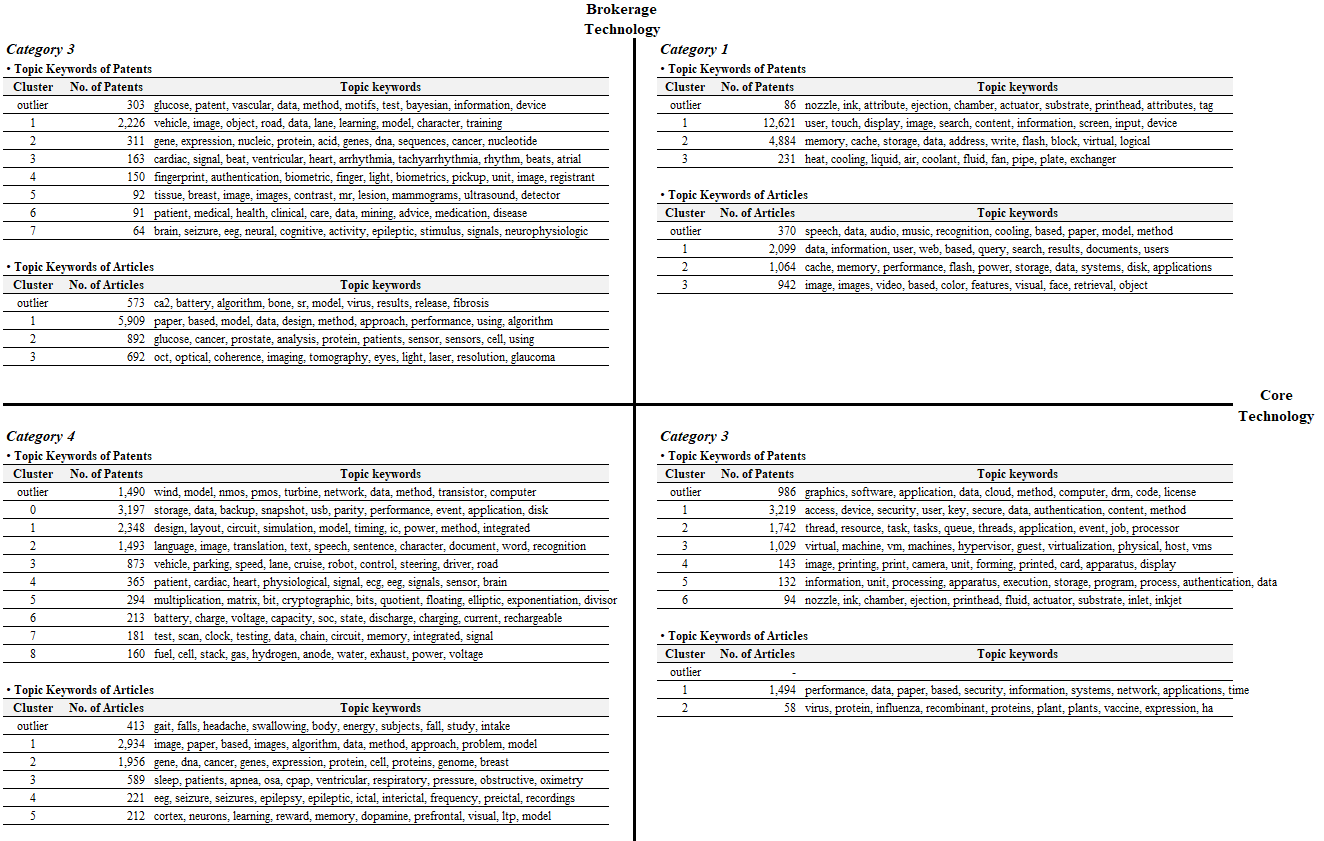


**Period 3 (2012-2016)**


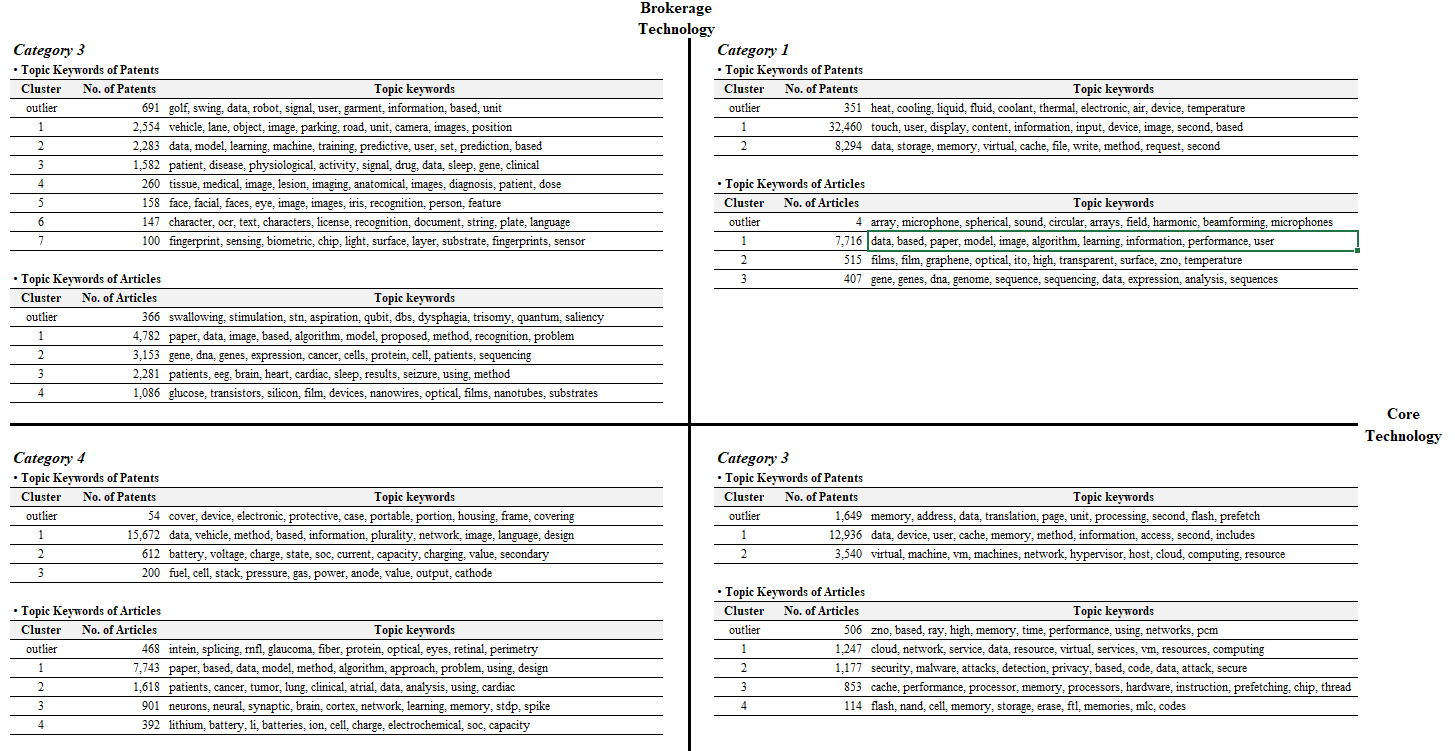


**Period 4 (2017-2021)**


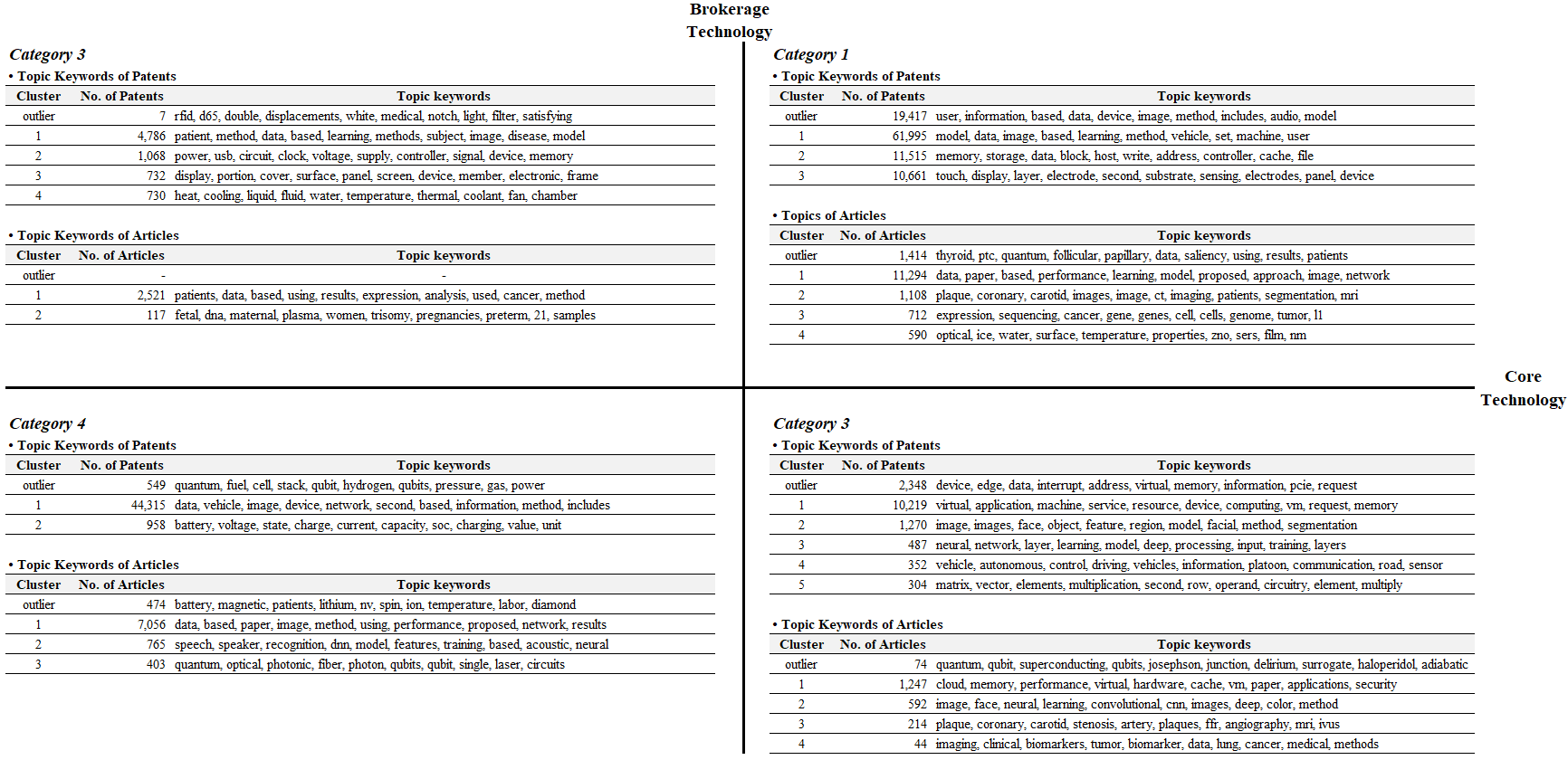

Supplement: S2 Fig — (DOCX) [file pone.0341005.s002.docx]

**S3 Figure. Topics from label generation**

**Period 1 (2002-2006)**


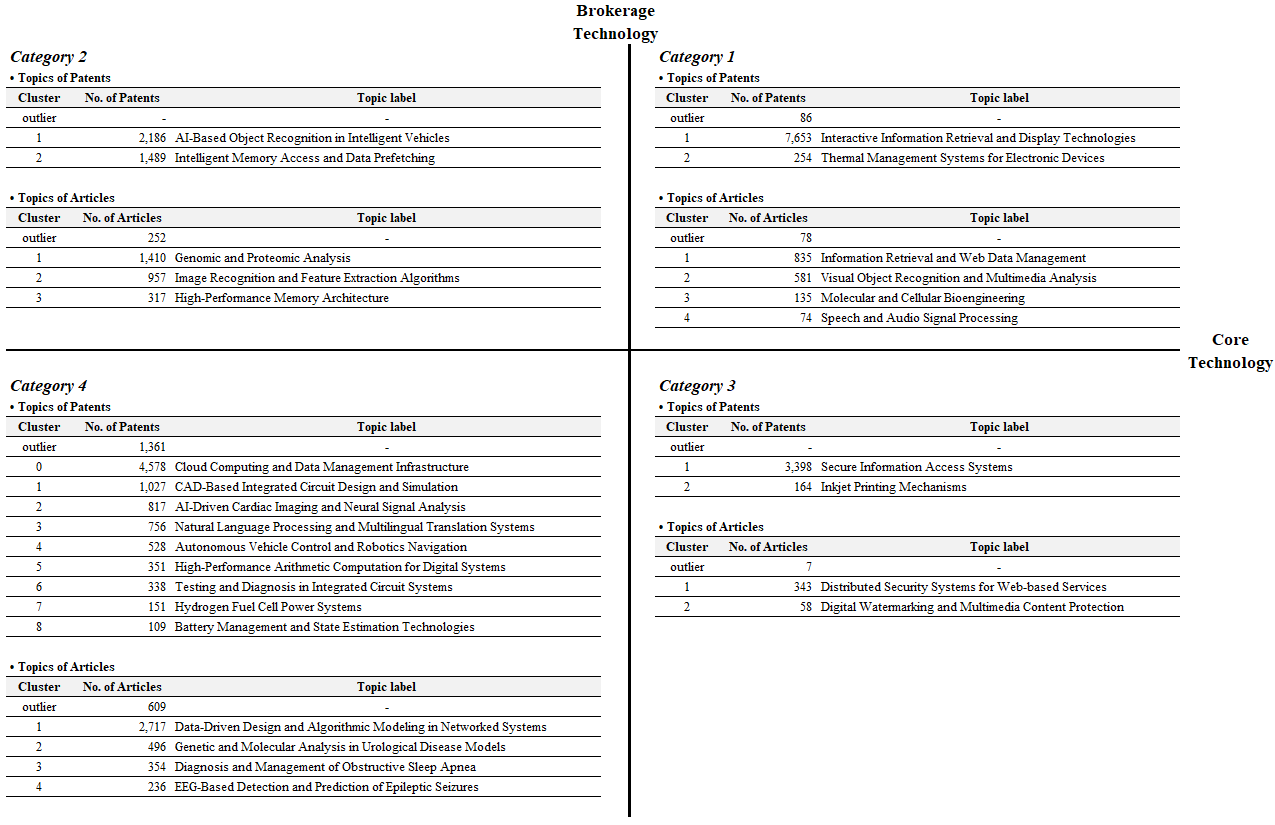


**Period 2 (2007-2011)**


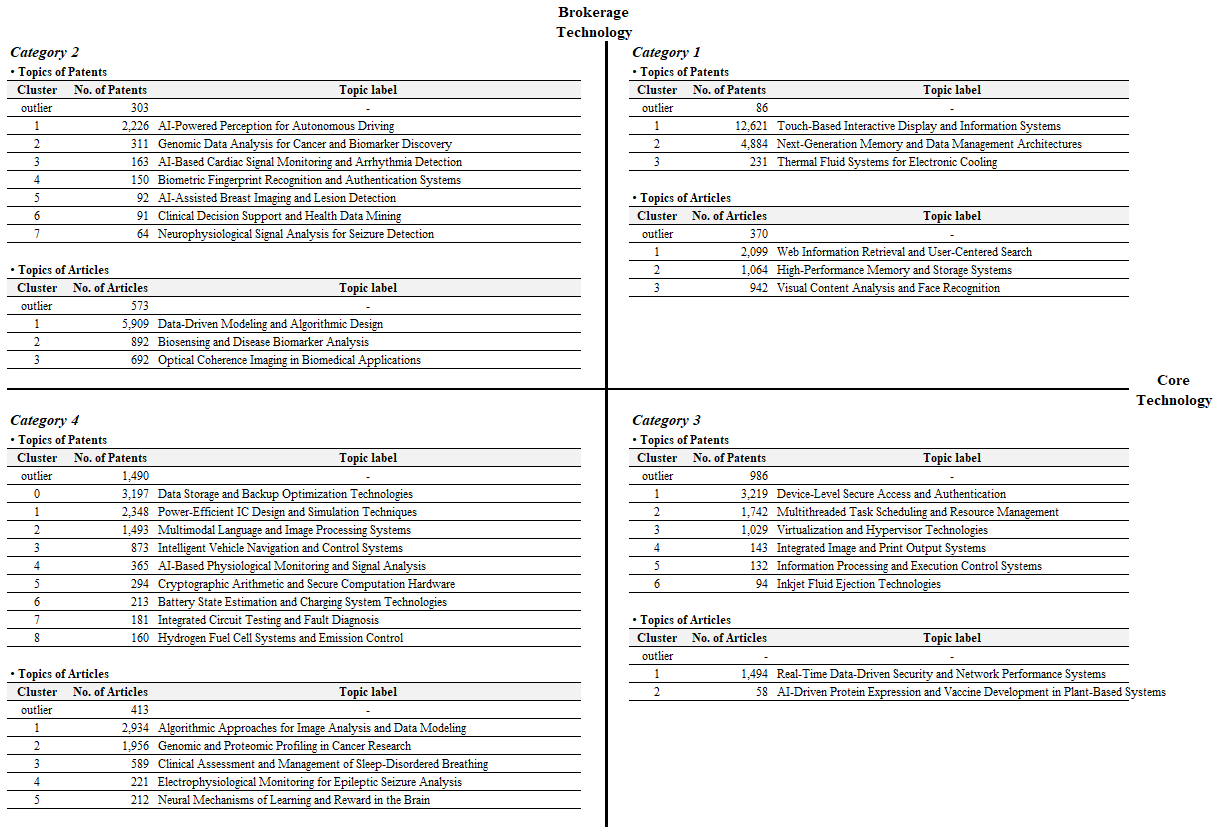


**Period 3 (2012-2016)**

**
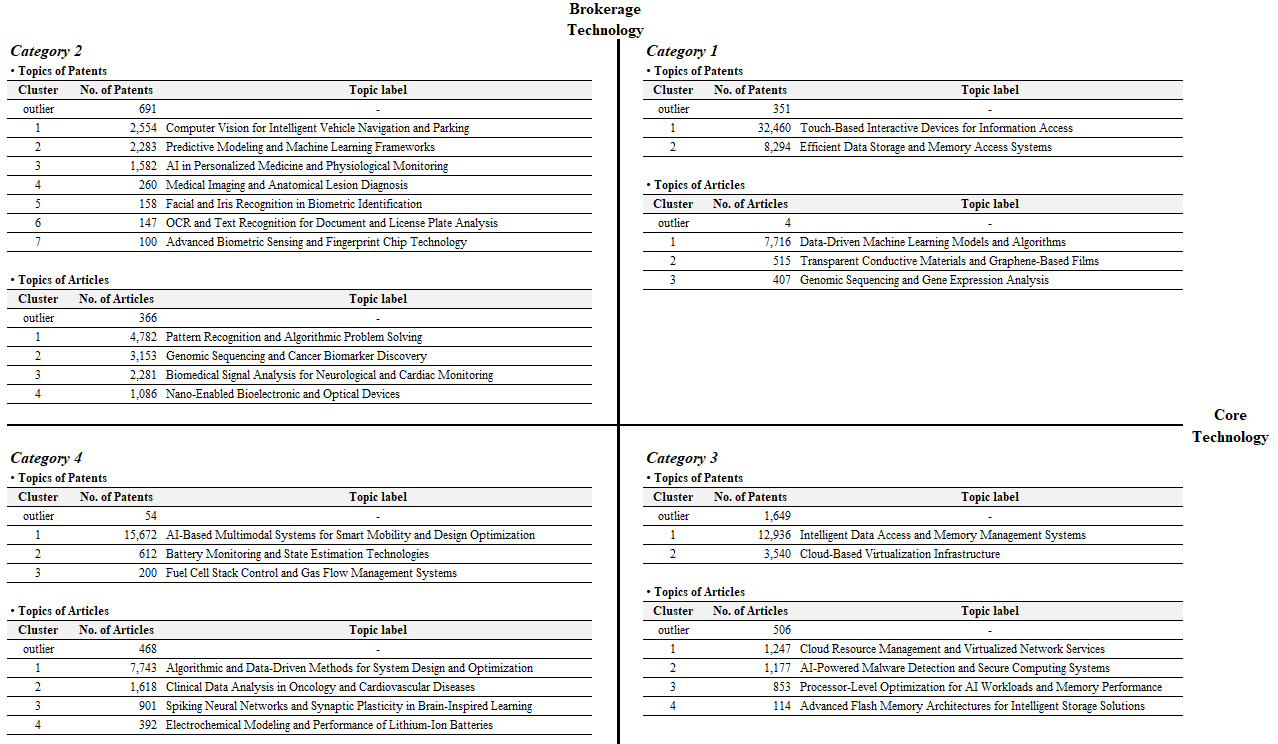
**

**Period 4 (2017-2021)**

**
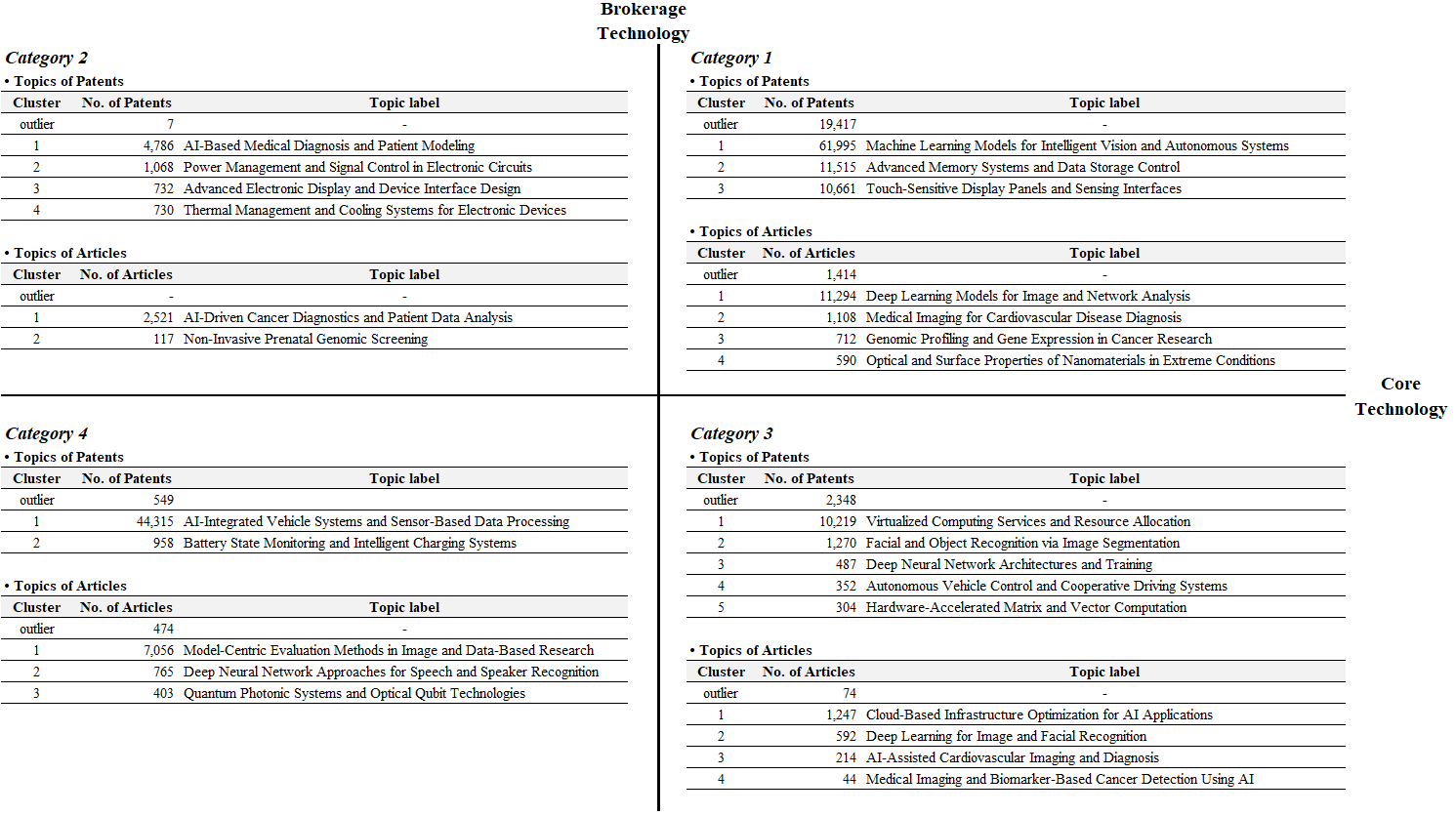
**

Supplement: S3 Fig — (DOCX) [file pone.0341005.s003.docx]

# **S4 Figure. Topic keywords from BERTopic modeling (10-years periods)**

**Period 1 (2002-2011)**

**
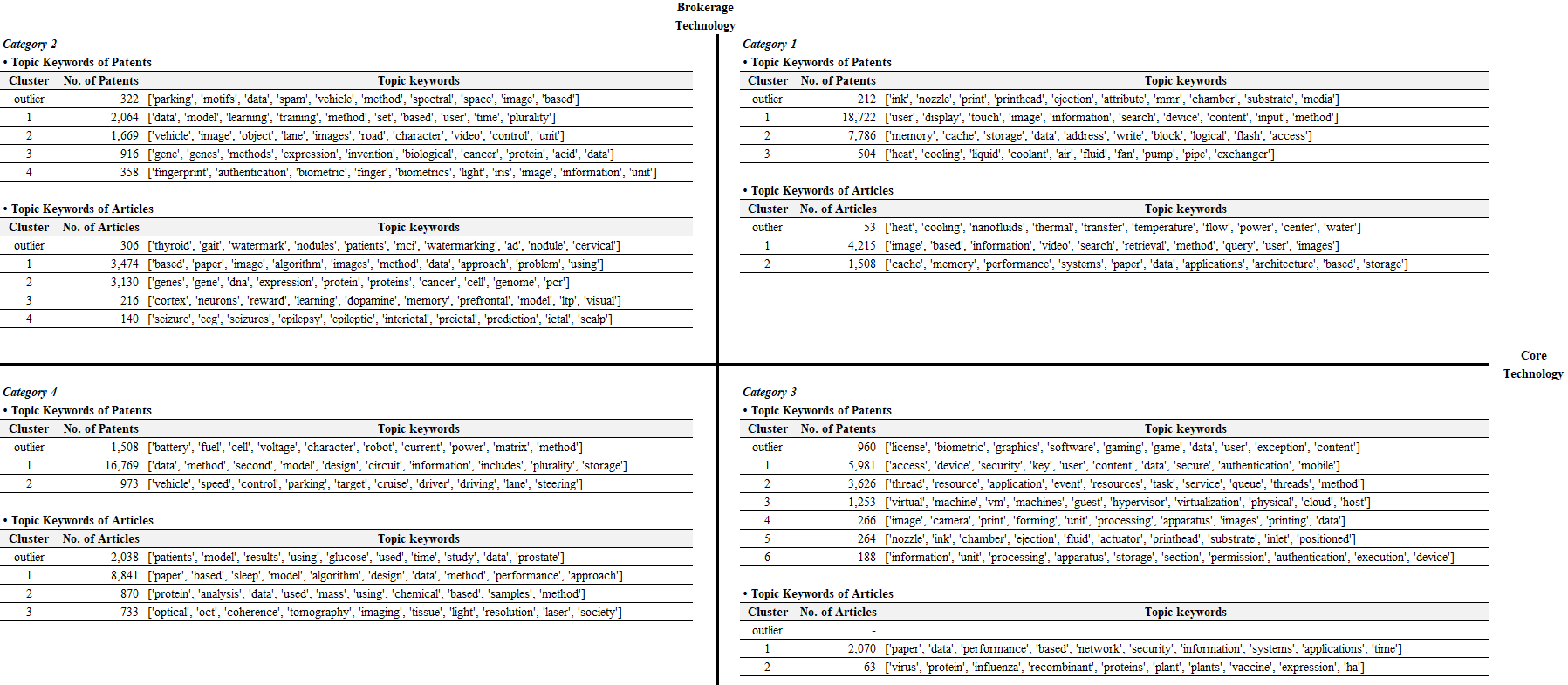
**

**Period 2 (2012-2021)**

**
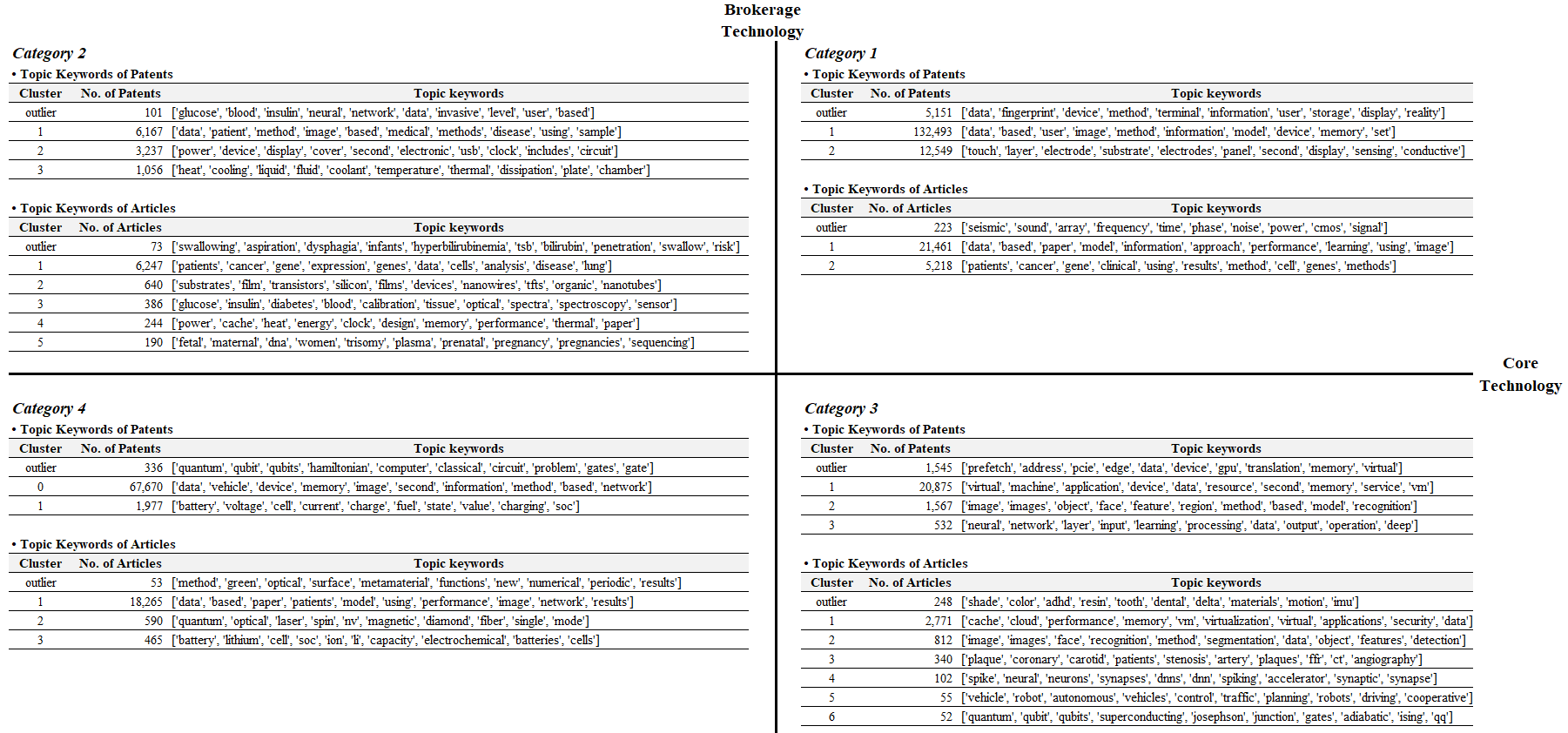
**

Supplement: S4 Fig — (DOCX) [file pone.0341005.s004.docx]

**S5 Figure. Topics from label generation (10-years periods)**

**Period 1 (2002-2011)**

**
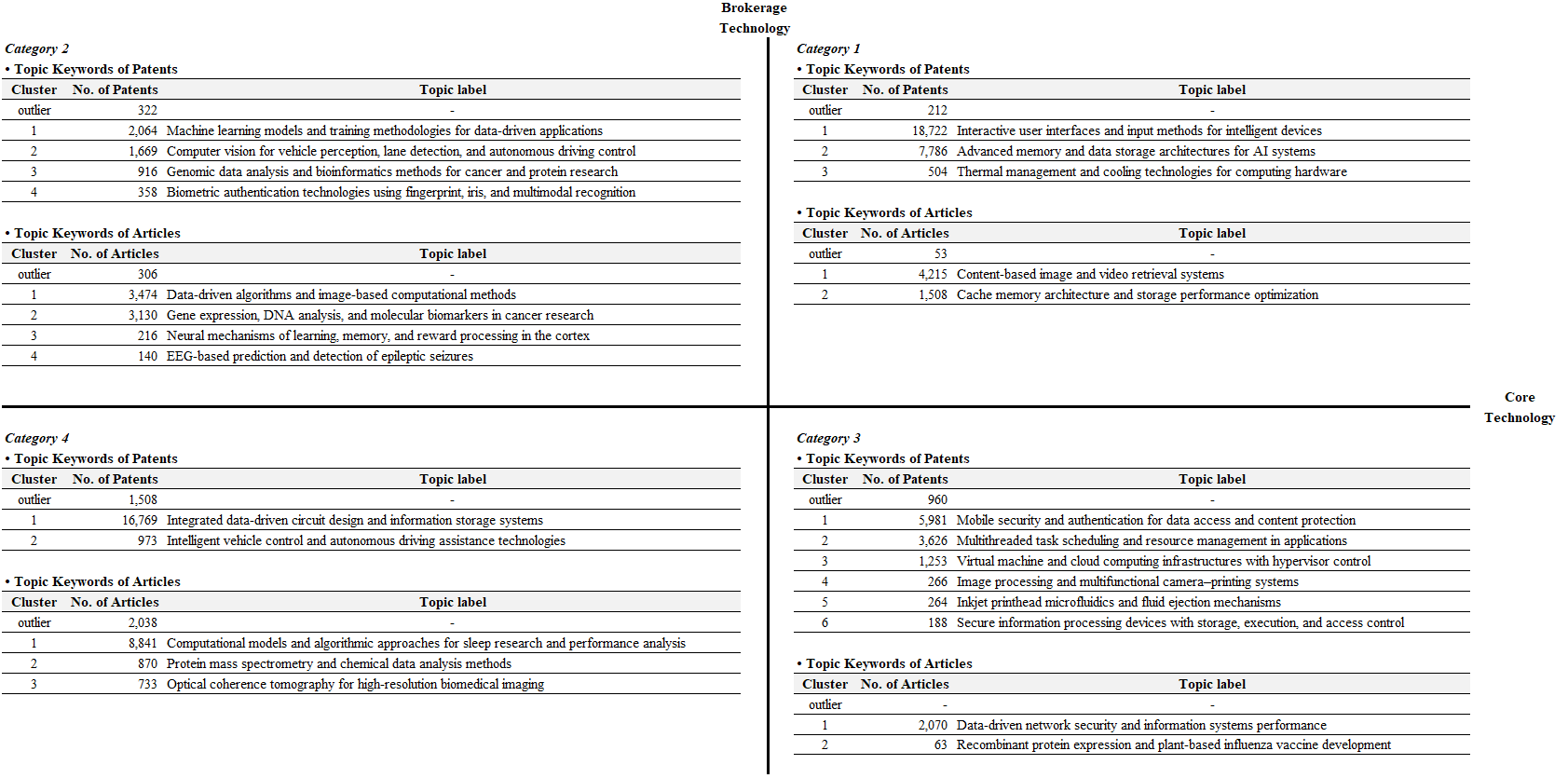
**

**Period 2 (2012-2021)**

**
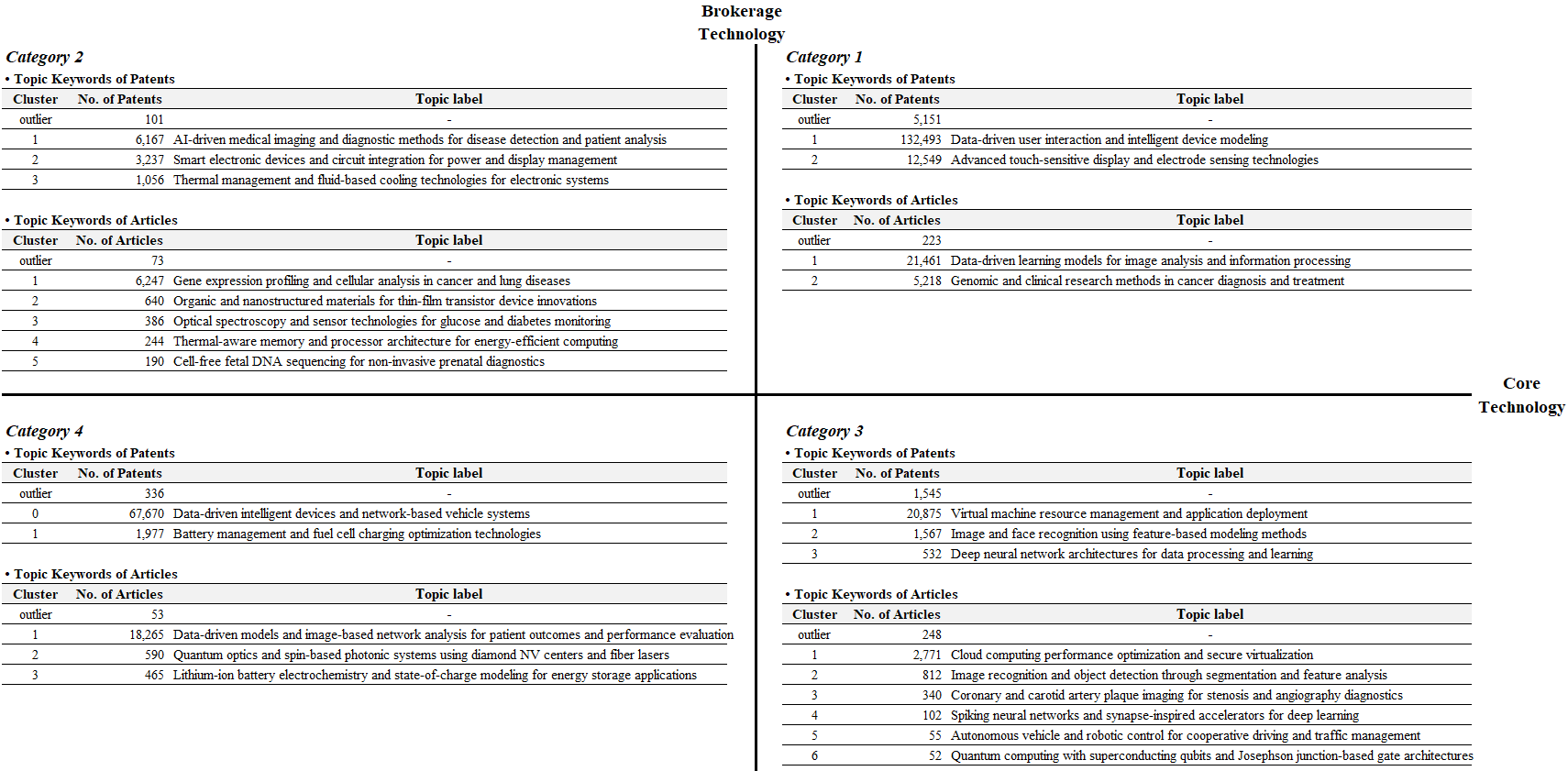
**

Supplement: S5 Fig — (DOCX) [file pone.0341005.s005.docx]
