# Supplementary material for: Knowledge flows from science to AI technology: Identifying core and brokerage technological roles
Source: PLoS One. 2026 Feb 19;21(2):e0341005. doi: 10.1371/journal.pone.0341005 (PMC12919798; doi:10.1371/journal.pone.0341005)
Supplement: S6 Fig — (DOCX) [file pone.0341005.s006.docx]

**S6 Figure. Representative patents and their cited scientific publications by category (Period 4: 2017–2021)**

**Category 1.**

**
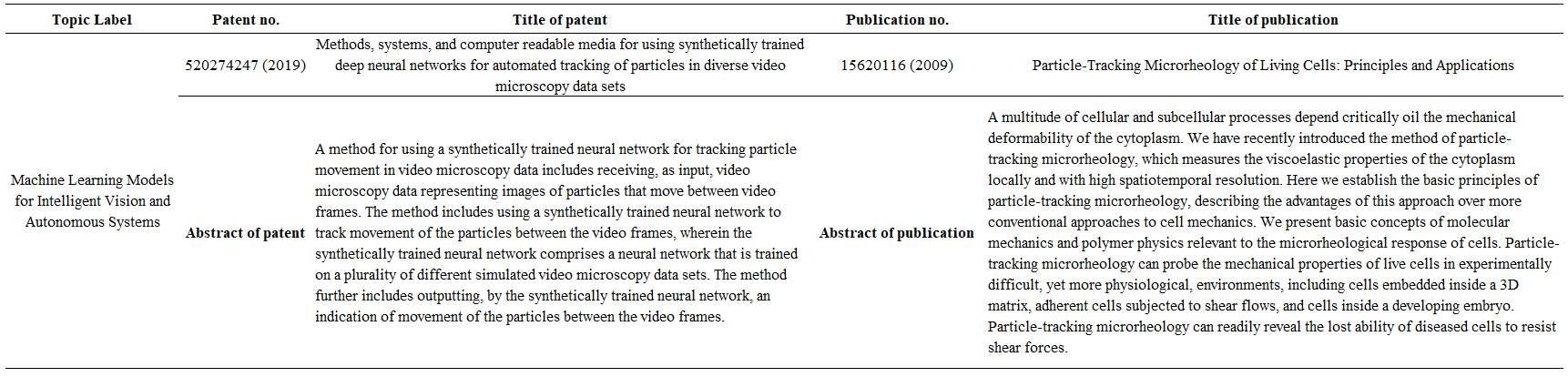
**

**Category 2.**

**
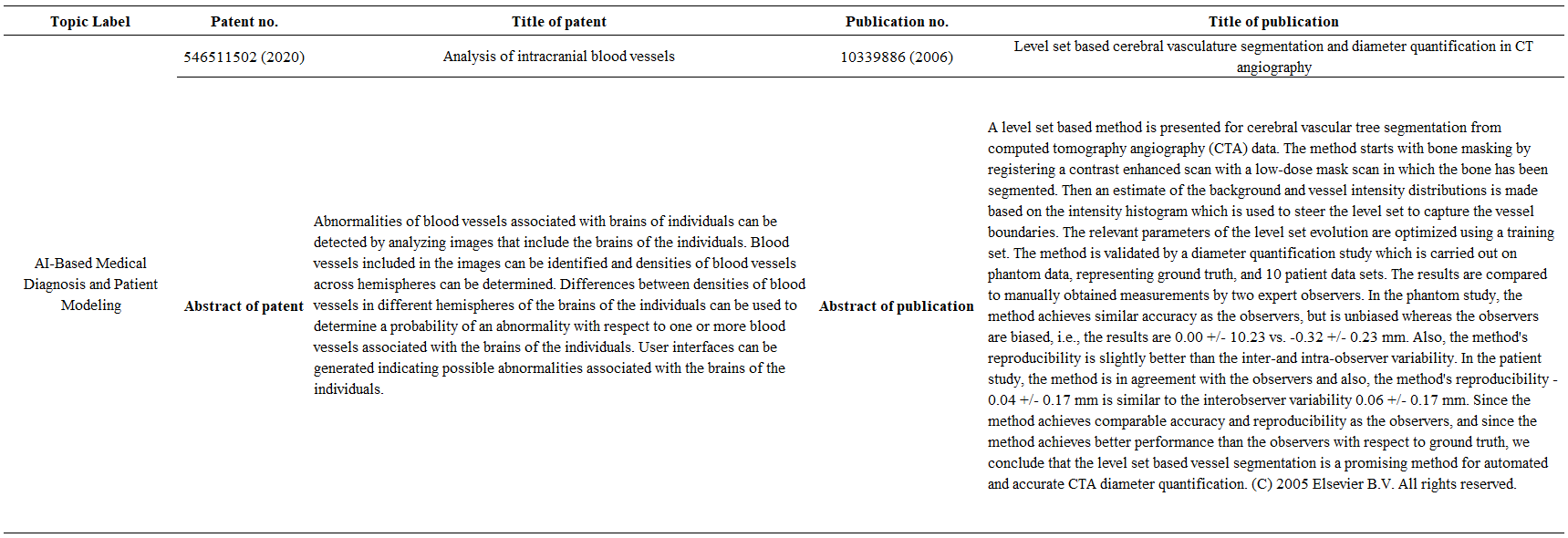
**

**Category 3.**

**
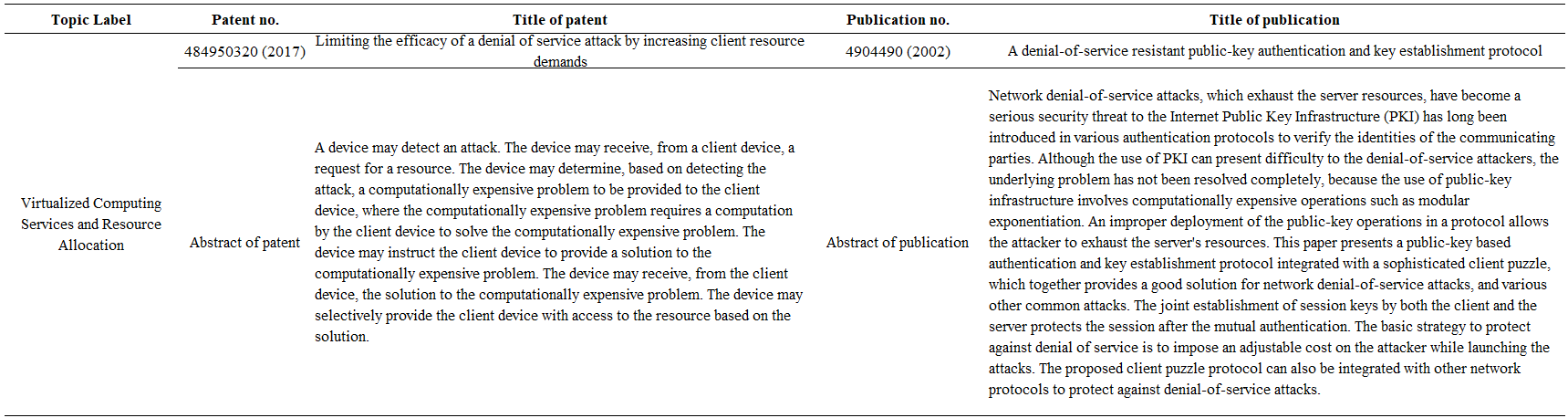
**

**Category 4.**

**
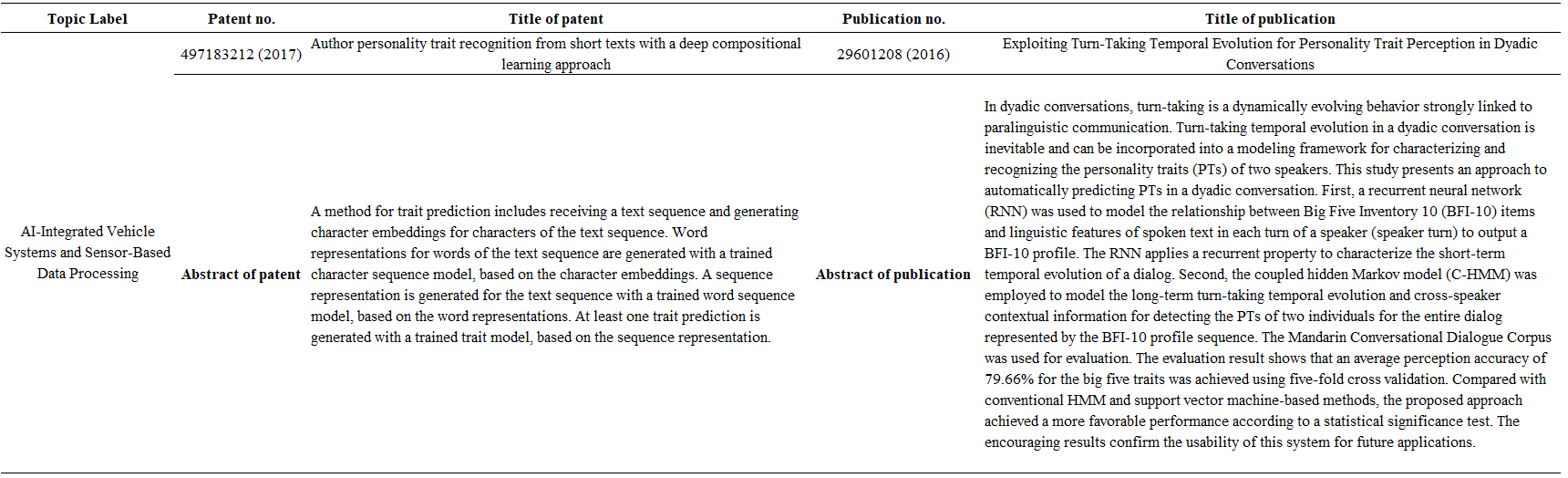
**
