# Supplementary material for: Knowledge flows from science to AI technology: Identifying core and brokerage technological roles
Source: PLoS One. 2026 Feb 19;21(2):e0341005. doi: 10.1371/journal.pone.0341005 (PMC12919798; doi:10.1371/journal.pone.0341005)
Supplement: S7 Table — (DOCX) [file pone.0341005.s007.docx]

**S7 Table. Descriptive statistics and correlations for additional regression analysis variables**

| Variables | | [1] | [2] | [3] | [4] | [5] | [6] | [7] |
| --- | --- | --- | --- | --- | --- | --- | --- | --- |
| [1] | *Number. of cited publications* | 1 |  |  |  |  |  |  |
| [2] | *CLAIMS* | 0.0875 | 1 |  |  |  |  |  |
| [3] | *FAMILY* | 0.0316 | 0.0032 | 1 |  |  |  |  |
| [4] | *INVENTORS* | 0.0178 | 0.0264 | 0.0197 | 1 |  |  |  |
| [5] | *APPLICANTS* | 0.0530 | 0.1420 | 0.0016 | 0.1417 | 1 |  |  |
| [6] | *PAT AGE* | 0.0612 | 0.1905 | 0.0162 | -0.1239 | 0.3733 | 1 |  |
| [7] | *CATEGORY dummy* |  |  |  |  |  |  |  |
| Min | | 0.000 | 0.000 | 0.393 | 0.693 | 0.000 | 0.000 | 0.000 |
| Max | | 609.000 | 5.903 | 6.136 | 4.159 | 4.143 | 2.996 | 3.000 |
| Mean | | 0.424 | 1.905 | 1.324 | 1.295 | 0.858 | 1.537 | 1.085 |
| S.D. | | 3.618 | 1.399 | 0.620 | 0.453 | 0.359 | 0.866 | 0.934 |

**Note: N = 319,597 patent-level observations**
