# Supplementary material for: Knowledge flows from science to AI technology: Identifying core and brokerage technological roles
Source: PLoS One. 2026 Feb 19;21(2):e0341005. doi: 10.1371/journal.pone.0341005 (PMC12919798; doi:10.1371/journal.pone.0341005)
Supplement: S1 File — (DOCX) [file pone.0341005.s008.docx]

# **Supporting Information**

# **S1 Appendix. Query strategies for AI patents**

**Full Query = Query Block 1 OR Query Block 2 OR Query Block 3**

**Query Block 1 (CPC Subgroups):**

(A61B 5/7264:A61B 5/7267 OR A61B 5/7267 OR A63F13/67 OR B23K 31/006 OR B25J 9/161 OR B29C 66/965 OR B29C2945/76979 OR B60G2600/1876 OR B60G2600/1878 OR B60G2600/1879 OR B60W 30/06 OR B60W 30/10:B60W 30/12 OR B60W 30/14:B60W 30/17 OR B62D 15/0285 OR B64G2001/247 OR E21B2041/0028 OR F02D 41/1405 OR F03D 7/046 OR F05B2270/707 OR F05B2270/709 OR F05D2270/709 OR F16H2061/0081 OR F16H2061/0084 OR G01N 29/4481 OR G01N 33/0034 OR G01N2201/1296 OR G01R 31/2846:G01R 31/2848 OR G01R 31/3651 OR G01S 7/417 OR G05B 13/027 OR G05B 13/0275 OR G05B 13/028 OR G05B 13/0285 OR G05B 13/029 OR G05B 13/0295 OR G05B2219/33002 OR G05D 1 OR G05D 1/0088 OR G06F 11/1476 OR G06F 11/2257 OR G06F 11/2263 OR G06F 15/18 OR G06F 17/16 OR G06F 17/2282 OR G06F 17/27:G06F 17/2795 OR G06F 17/28:G06F 17/289 OR G06F 17/30029:G06F 17/30035 OR G06F 17/30247:G06F 17/30262 OR G06F 17/30401 OR G06F 17/3043 OR G06F 17/30522:G06F 17/3053 OR G06F 17/30654 OR G06F 17/30663 OR G06F 17/30666 OR G06F 17/30669 OR G06F 17/30672 OR G06F 17/30684 OR G06F 17/30687 OR G06F 17/3069 OR G06F 17/30702 OR G06F 17/30705:G06F 17/30713 OR G06F 17/30731:G06F 17/30737 OR G06F 17/30743:G06F 17/30746 OR G06F 17/30784:G06F 17/30814 OR G06F 19/24 OR G06F 19/707 OR G06F2207/4824 OR G06K 7/1482 OR G06K 9 OR G06N 3 OR G06N 3/004:G06N 3/008 OR G06N 5/003:G06N 5/027 OR G06N 7/005:G06N 7/06 OR G06N 7/046 OR G06N 99/005 OR G06T 3/4046 OR G06T 9/002 OR G06T2207/20081 OR G06T2207/20084 OR G06T2207/20084 OR G06T2207/30236 OR G06T2207/30248:G06T2207/30268 OR G08B 29/186 OR G10H2250/151 OR G10H2250/311 OR G10K2210/3024 OR G10K2210/3038 OR G10L 15 OR G10L 17 OR G10L 25/30 OR G11B 20/10518 OR H01J2237/30427 OR H01M 8/04992 OR H02H 1/0092 OR H02P 21/0014 OR H02P 23/0018 OR H03H2017/0208 OR H03H2222/04 OR H04L 25/0254 OR H04L 25/03165 OR H04L 41/16 OR H04L 45/08 OR H04L2012/5686 OR H04L2025/03464 OR H04L2025/03554 OR H04N 21/4662:H04N 21/4666 OR H04N 21/4666 OR H04Q2213/054 OR H04Q2213/13343 OR H04Q2213/343 OR H04R 25/507 OR Y10S 128/924 OR Y10S 128/925 OR Y10S 706)

**Query Block 2 (Keywords):**

(ARTIFICIAL INTELLIGEN+ OR COMPUTATIONAL INTELLIGEN+ OR NEURAL_NETWORK+ OR BAYESIAN_NETWORK+ OR CHATBOT+ OR DATA_MINING+ OR DECISION_MODEL+ OR DEEP_LEARNING+ OR GENETIC_ALGORITHM+ OR INDUCTIVE LOGIC PROGRAMM+ OR MACHINE_LEARNING+ OR NATURAL LANGUAGE GENERATION+ OR NATURAL LANGUAGE PROCESSING+ OR REINFORCEMENT_LEARNING+ OR SUPERVISED_LEARNING+ OR SUPERVISED TRAINING+ OR SWARM_INTELLIGEN+ OR UNSUPERVISED_LEARNING+ OR UNSUPERVISED TRAINING+ OR SEMI_SUPERVISED_LEARNING+ OR SEMI_SUPERVISED TRAINING+ OR CONNECTIONIS+ OR EXPERT SYSTEM+ OR FUZZY LOGIC+ OR TRANSFER_LEARNING+ OR LEARNING ALGORITHM+ OR LEARNING MODEL+ OR SUPPORT VECTOR MACHINE+ OR RANDOM FOREST+ OR DECISION TREE+ OR GRADIENT TREE BOOSTING+ OR XGBOOST+ OR ADABOOST+ OR RANKBOOST+ OR LOGISTIC REGRESSION+ OR STOCHASTIC GRADIENT DESCENT+ OR MULTI-LAYER PERCEPTRON+ OR LATENT SEMANTIC ANALYSIS+ OR LATENT DIRICHLET ALLOCATION+ OR MULTI-AGENT SYSTEM+ OR HIDDEN MARKOV MODEL+)

**Query Block 3 (IPC Subgroups + CPC Subgroups + Keywords):**

**Query Block 3 = ((Q3.1 OR Q3.2) AND Q3.3)**

**Q3.1 = IPC** (A61B 5 OR A63F 13/67 OR B23K 31 OR B25J 9/16:B25J 9/20 OR B29C 65 OR B60W 30/06 OR B60W 30/10:B60W 30/12 OR B60W 30/14:B60W 30/17 OR B62D 15/02:B62D 15/0295 OR B64G 1/24:B64G 1/38 OR E21B 41 OR F02D 41/14:F02D 41/16 OR F03D 7/04:F03D 7/048 OR F16H 61 OR G01N 29/44:G01N 29/52 OR G01N 33 OR G01R 31/28:G01R 31/31937 OR G01R 31/36:G01R 31/3696 OR G01S 7/41:G01S 7/418 OR G05B 13/02:G05B 13/048 OR G05D 1 OR G06F 9/44+ OR G06F 11/14:G06F 11/1497 OR G06F 11/22:G06F 11/277 OR G06F 15/18 OR G06F 17/14 OR G06F 17/15 OR G06F 17/16 OR G06F 17/20 OR G06F 17/27 OR G06F 17/28 OR G06F 19/24 OR G06F 21 OR G06K 7/14:G06K 7/1495 OR G06K 9 OR G06N 3 OR G06N 5 OR G06N 7 OR G06N 99 OR G06T 1/20 OR G06T 1/40+ OR G06T 3/40:G06T 3/4092 OR G06T 7 OR G06T 9 OR G07C 9 OR G08B 29/18:G08B 29/28 OR G10L 13 OR G10L 15 OR G10L 17 OR G10L 25 OR G10L 99 OR G11B 20/10:G11B 20/18 OR G16H 50/20 OR H01M 8/04992 OR H02H 1 OR H02P 21 OR H02P 23 OR H03H 17/02:H03H 17/06 OR H04L 12/24+ OR H04L 12/70+ OR H04L 12/751+ OR H04L 25/02:H04L 25/26 OR H04L 25/03:H04L 25/03993 OR H04N 21/466:H04N 21/4668 OR H04R 25)

**Q3.2 = CPC** (G06F 17/14:G06F 17/148 OR G06F 17/153 OR G06F 21 OR G06F 30/00,:G06F 30/12, OR G06F2111/00:G06F2111/20 OR G06F2113/00:G06F2113/28 OR G06F2115/00:G06F2115/12 OR G06F2117/00:G06F2117/12 OR G06F2119/00:G06F2119/22 OR G06Q 30/02:G06Q 30/0284 OR G06T 1/20 OR G06T 7 OR G07C 9 OR G10H2250/005:G10H2250/021 OR G10L 13 OR G10L 25 OR G10L 99)

**Q3.3 = Keywords** (CLUSTERING+ OR COMPUTATIONAL CREATIVITY+ OR DESCRIPTIVE MODEL+ OR INDUCTIVE REASONING+ OR OVERFITTING+ OR PREDICTIVE ANALYTICS+ OR PREDICTIVE MODEL+ OR TARGET FUNCTION+ OR TEST DATA+ OR TRAINING DATA+ OR VALIDATION DATA+ OR BACKPROPAGATION+ OR SELF_LEARNING+ OR OBJECTIVE FUNCTION+ OR FEATURE SELECTION+ OR EMBEDDING+ OR ACTIVE LEARNING+ OR REGRESSION MODEL+ OR STOCHASTIC APPROACH+ OR STOCHASTIC TECHNIQUE+ OR STOCHASTIC METHOD+ OR STOCHASTIC ALGORITHM+ OR PROBABILISTIC APPROACH+ OR PROBABILISTIC TECHNIQUE+ OR PROBABILISTIC METHOD+ OR PROBABILISTIC ALGORITHM+ OR RECOMMENDATION SYSTEM+ OR TEXT ANALY+ OR TEXT RECOGNITION+ OR SPEECH ANALY+ OR SPEECH RECOGNITION+ OR HAND_WRITING ANALY+ OR HAND_WRITING RECOGNITION+ OR FACIAL ANALY+ OR FACIAL RECOGNITION+ OR FACE ANALY+ OR FACE RECOGNITION+ OR CHARACTER ANALY+ OR CHARACTER RECOGNITION+)

# **S2 Appendix. Topic keywords from BERTopic modeling**

**Period 1 (2002-2006)**

**Period 2 (2007-2011)**

**Period 3 (2012-2016)**

**Period 4 (2017-2021)**

**S3 Appendix. Topics from label generation**

**Period 1 (2002-2006)**

**Period 2 (2007-2011)**

**Period 3 (2012-2016)**

**Period 4 (2017-2021)**

# **S4 Appendix. Topic keywords from BERTopic modeling (10-years periods)**

**Period 1 (2002-2011)**

**Period 2 (2012-2021)**

**S5 Appendix. Topics from label generation (10-years periods)**

**Period 1 (2002-2011)**

**Period 2 (2012-2021)**

**S6 Appendix. Representative patents and their cited scientific publications by category (Period 4: 2017–2021)**

**Category 1.**

**Category 2.**

**Category 3.**

**Category 4.**

**S7 Appendix. Descriptive statistics and correlations for additional regression analysis variables**

| Variables | | [1] | [2] | [3] | [4] | [5] | [6] | [7] |
| --- | --- | --- | --- | --- | --- | --- | --- | --- |
| [1] | *Number. of cited publications* | 1 |  |  |  |  |  |  |
| [2] | *CLAIMS* | 0.0875 | 1 |  |  |  |  |  |
| [3] | *FAMILY* | 0.0316 | 0.0032 | 1 |  |  |  |  |
| [4] | *INVENTORS* | 0.0178 | 0.0264 | 0.0197 | 1 |  |  |  |
| [5] | *APPLICANTS* | 0.0530 | 0.1420 | 0.0016 | 0.1417 | 1 |  |  |
| [6] | *PAT AGE* | 0.0612 | 0.1905 | 0.0162 | -0.1239 | 0.3733 | 1 |  |
| [7] | *CATEGORY dummy* |  |  |  |  |  |  |  |
| Min | | 0.000 | 0.000 | 0.393 | 0.693 | 0.000 | 0.000 | 0.000 |
| Max | | 609.000 | 5.903 | 6.136 | 4.159 | 4.143 | 2.996 | 3.000 |
| Mean | | 0.424 | 1.905 | 1.324 | 1.295 | 0.858 | 1.537 | 1.085 |
| S.D. | | 3.618 | 1.399 | 0.620 | 0.453 | 0.359 | 0.866 | 0.934 |

**Note: N = 319,597 patent-level observations**
